# Delivery of Active AKT1 to Human Cells

**DOI:** 10.3390/cells11233834

**Published:** 2022-11-29

**Authors:** Tarana Siddika, Nileeka Balasuriya, Mallory I. Frederick, Peter Rozik, Ilka U. Heinemann, Patrick O’Donoghue

**Affiliations:** 1 Department of Biochemistry, The University of Western Ontario, London, ON N6A 5C1, Canada; 2 Department of Chemistry, The University of Western Ontario, London, ON N6A 5C1, Canada

**Keywords:** cell penetrating peptide, cellular signaling, kinase, protein kinase B (AKT1), phosphoinositide-dependent kinase (PDK1), recombinant protein, trans-activator of transcription (TAT)

## Abstract

Protein kinase B (AKT1) is a serine/threonine kinase and central transducer of cell survival pathways. Typical approaches to study AKT1 biology in cells rely on growth factor or insulin stimulation that activates AKT1 via phosphorylation at two key regulatory sites (Thr308, Ser473), yet cell stimulation also activates many other kinases. To produce cells with specific AKT1 activity, we developed a novel system to deliver active AKT1 to human cells. We recently established a method to produce AKT1 phospho-variants from *Escherichia coli* with programmed phosphorylation. Here, we fused AKT1 with an N-terminal cell penetrating peptide tag derived from the human immunodeficiency virus trans-activator of transcription (TAT) protein. The TAT-tag did not alter AKT1 kinase activity and was necessary and sufficient to rapidly deliver AKT1 protein variants that persisted in human cells for 24 h without the need to use transfection reagents. TAT-pAKT1^T308^ induced selective phosphorylation of the known AKT1 substrate GSK-3α, but not GSK-3β, and downstream stimulation of the AKT1 pathway as evidenced by phosphorylation of ribosomal protein S6 at Ser240/244. The data demonstrate efficient delivery of AKT1 with programmed phosphorylation to human cells, thus establishing a cell-based model system to investigate signaling that is dependent on AKT1 activity.

## 1. Introduction

**Figure 1:**
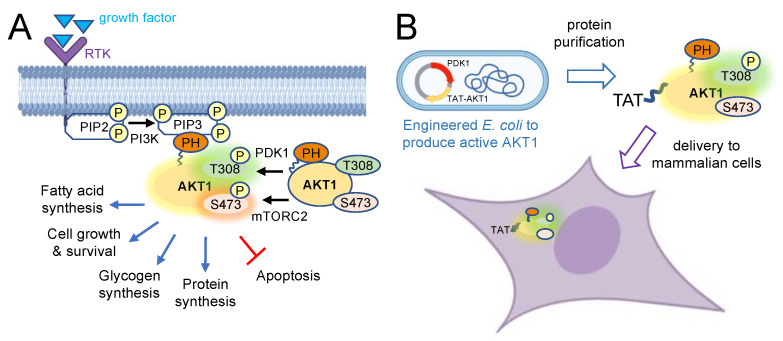
AKT1 activation pathway and delivery of site-specifically phosphorylated AKT1 into human cells. (**A**) Protein kinase B (AKT1) is normally activated in response to growth factors that bind to a receptor tyrosine kinase (RTK) in the membrane, activating phosphoinositide 3-kinase (PI3K). PI3K subsequently phosphorylates phosphatidylinositol-4,5-bisphosphate (PIP2) to phosphatidylinositol-3,4,5-trisphosphate (PIP3). When the pleckstrin homology (PH) domain of AKT1 binds to the membrane phospholipid PIP3, the auto-inhibitory effect of the PH domain is released from AKT1, exposing the activation sites of AKT1 for phosphorylation by the up-stream kinases mTOR complex 2 (mTORC2) at Ser473 and phosphoinositide-dependent kinase 1 (PDK1) phosphorylates Thr308. The activation of AKT1 initiates a phosphorylation cascade, leading to the activation of many downstream pathways controlling cell growth and protein synthesis as well as inhibition of apoptosis. (**B**) Trans-Activator of Transcription (TAT) is a cell penetrating peptide facilitating protein delivery to mammalian cells. TAT-tagged AKT1 was expressed and purified from *E. coli* cells. PDK1 was co-expressed in *E. coli* to facilitate phosphorylation of AKT1 at Thr308 during protein production, yielding active, phosphorylated TAT-tagged AKT1. TAT-tagged AKT1 variants were then delivered to human cells.

Protein kinase B (PKB or AKT1) is a serine/threonine kinase and a key mediator of cell growth and survival processes, including glucose metabolism, apoptosis, transcription, cell proliferation, and migration [1,2]. The AKT protein family encompasses three isoforms: AKT1, AKT2, and AKT3 [3]. The AKT isoforms have both overlapping substrates and unique functions that are not compensated for by the other isoforms due to their individual subcellular localization [4]. Overexpression of AKT isoforms is associated with multiple human cancers [5,6,7], and AKT1 is a prime drug target [8] that is hyper-phosphorylated and overactive in most human cancers [9]. AKT1 is activated by phosphorylation at two key regulatory sites (Thr308 and Ser473) that are both used as clinical diagnostic markers [10]. Activated AKT1 phosphorylates downstream targets that stimulate cell survival and inhibit apoptosis [11] (Figure 1). Thr308 and Ser473 phosphorylation is also regulated by phosphatases. Protein phosphatase 2A (PP2A) dephosphorylates AKT1 at Thr308, and PH domain leucine-rich repeat phosphatase (PHLPP) dephosphorylates Ser473 [12,13].

AKT1 contains three conserved domains, namely the N-terminal pleckstrin homology domain (PH), followed by the central kinase catalytic (CAT) domain and the C-terminal extension (EXT) domain containing the hydrophobic motif (HM) and proline-rich sequences [14,15]. The PH domain plays an important role in co-localization with upstream kinases and translocation of AKT1 from the cytoplasm to the cell membrane where it is activated. In response to growth factor, the phosphoinositide 3-kinase (PI3K) phosphorylates phosphatidylinositol (4,5)-bisphosphate (PIP2) to generate phosphatidylinositol (3,4,5)-trisphosphate (PIP3). As a result, PH domain-containing proteins, including AKT1, bind to PIP3 and transiently anchor to the plasma membrane [16,17].

At the cell membrane, another PH-domain containing protein, phosphoinositide-dependent kinase 1 (PDK1) binds to PIP3 and phosphorylates AKT1 in the catalytic domain at Thr308 [18]. Activated by receptor tyrosine kinases (RTK), mammalian target of rapamycin complex 2 (mTORC2) phosphorylates AKT1 in the HM domain at Ser473 [15] (Figure 1). Fully activated AKT1 has many downstream substrates [19]. Phosphorylated AKT1 plays an important role in glucose metabolism by inhibiting glycogen synthase kinase-3 (GSK-3) isoforms through phosphorylation of GSK-3α at Ser21 and GSK-3β at Ser9 [20,21]. AKT1-dependent phosphorylation of the tuberous sclerosis 2 (TSC2) protein counteracts its inhibitory effect on mammalian target of rapamycin complex 1 (mTORC1), a regulator of protein synthesis that affects many cellular processes including cell survival and growth. Stimulated mTORC1 activates the p70 ribosomal protein S6 kinase which leads to phosphorylation of ribosomal protein S6 at Ser240/244 [22,23,24] and stimulation of protein synthesis [8].

Traditional approaches to investigate AKT1 biology are based on the over-expression of AKT1 followed by stimulation of cells with insulin or epidermal growth factor (EGF) leading to phosphorylation and activation of AKT1 [25,26]. EGF treatment activates AKT1 as well as Raf-1 kinase [27], mitogen activated protein kinases [28], and many other protein kinases and signaling cascades [29,30]. We previously developed an approach to generate recombinant and active human AKT1 from bacterial cells with site-specific phosphorylation [19,31,32,33,34]. Here, we fused the cell penetrating TAT peptide to recombinant AKT1 constructs and produced and purified both inactive (TAT-AKT1) and active (TAT-pAKT1^T308^) AKT1 variants. We demonstrated the delivery of TAT-AKT1 and TAT-pAKT1^T308^ to human cells in culture. We found that active TAT-pAKT1^T308^ but not inactive TAT-AKT1 delivery led to increased phosphorylation of AKT1 targets and activated AKT1 signaling pathways in human cells.

## 2. Materials and Methods

### 2.1. Molecular Cloning and Gene Synthesis

The human AKT1 gene, which was acquired from the Harvard PlasmidID repository service (Boston, MA, USA), was previously cloned [33] using *Nco*I/*Not*I restriction sites into the pCDF-Duet-1 vector in multiple-cloning site 1 (MCS1). The unphosphorylated AKT1 was produced from this pCDF-Duet-1 vector, with no gene cloned into multiple-cloning site 2 (MCS2). For pAKT1^T308^ production, the MCS2 contains human PDK1 as described before [33]. Briefly, the human PDK1 gene, which was also purchased from the Harvard PlasmidID repository, was cloned using *Kpn*I/*Nde*I into MCS2 of pCDF-Duet-1 as detailed before. For TAT tagged gene constructs, the TAT-tag (YGRKKRRQRRR) and TAT-mCherry-tagged AKT1 constructs were codon optimized for *Escherichia coli* expression and synthesized (Azenta Life Sciences, Chelmsford, MA, USA). The His_6_-TAT tag DNA sequence was derived from the pTAT-HA vector, which was a kind gift from Steven Dowdy (Addgene, Watertown, MA, USA, plasmid #35612) [35]. The synthetic genes were flanked by *Nco*I/*Not*I restriction sites and cloned at Azenta Life Sciences into pCDF-Duet-1 and separately into the pCDF-Duet-1-PDK1 vector noted above. Each of the resulting AKT1 constructs contain a His_6_ tag for affinity purification. Sequences of each construct were verified by DNA sequencing at Azenta Life Sciences and are available in the Supplementary Material Section S.2.

### 2.2. Protein Production and Purification

All AKT1 protein variants were expressed in *E. coli* BL21(DE3) as His-tag fusion proteins and the six AKT1 variants (AKT1, pAKT1^T308^, TAT-AKT1, TAT-pAKT1^T308^, TAT-mCherry-AKT1, and TAT-mCherry-pAKT1^T308^) were purified by affinity chromatography followed by size exclusion and anion exchange chromatography (see below). For protein production, *E. coli* BL21(DE3) chemically competent cells were transformed with the indicated pCDF-Duet-1 vector and selected on Luria-Bertani (LB) agar with 50 µg/mL spectinomycin. Independent protein preparations were initiated with a single transformed *E. coli* colony that was used to inoculate 100 mL of liquid LB media with spectinomycin at 50 µg/mL and incubated overnight at 37 °C with shaking. From the starter culture, a 10 mL inoculum was added to 4 × 1 L of LB with spectinomycin at 50 µg/mL. The preparative cultures were grown with shaking at 37 °C until A_600_ = 0.8, when protein production was induced by adding 300 µM of isopropyl β-D-1-thiogalactopyranoside (IPTG). Bacterial cultures were then incubated at 16 °C for 18 h with shaking. Cells were pelleted by centrifugation at 5500× *g* and stored at −80 °C until further analysis. Cell pellets were suspended in lysis buffer (20 mM 4-(2-Hydroxyethyl)-1-piperazine ethanesulfonic acid (HEPES) pH 7.4, 150 mM NaCl, 5 mM β-mercaptoethanol, 1 mM phenylmethylsulfonyl fluoride (PMSF), 1 tablet of ethylenediaminetetraacetic acid (EDTA)-free mini protease inhibitor cocktail (Roche Canada, Mississauga, ON, Canada) and 10 mM imidazole). For the pAKT1^T308^ protein variants, phosphatase inhibitors (1 mM Na_3_VO_4_, 5 mM NaF) were added to the lysis buffer. Following sonication, cell lysates were centrifuged at 170,000× *g* for 1 h. The cell free extract was filtered using a sterile 0.45 µm filter.

### 2.3. Affinity Column Chromatography 

Ni^2+^-nitrilotriacetic acid (NTA) affinity column chromatography (HisTrap FF 5 mL column volume, #17525501, Sigma-Aldrich Canada, Oakville, ON, Canada) was used to purify all His-tagged AKT1 protein variants using an ÄKTA Pure L1 FPLC system (Cytiva Life Sciences, Shrewsbury, MA, USA). Columns were equilibrated with buffer A (20 mM HEPES pH 7.4, 150 mM NaCl, 5 mM β-mercaptoethanol, 15 mM imidazole), and cell lysates were loaded onto the column. Unbound proteins were washed with 10 column volumes of buffer A, and 8 column volumes of buffer B (20 mM HEPES pH 7.4, 150 mM NaCl, 5 mM β-mercaptoethanol, 30 mM imidazole). The His_6_-tagged proteins were eluted using 6 column volumes of elution buffer (20 mM HEPES pH 7.4, 150 mM NaCl, 5 mM β-mercaptoethanol, and 70 mM imidazole). The pAKT1^T308^ proteins were eluted in elution buffer containing 200 mM imidazole. Elution fractions of 1 mL were taken over the total 5 mL elution volume. Samples from each fraction were separated by 10% sodium dodecyl sulfate (SDS)-polyacrylamide gel electrophoresis (PAGE) and visualized with Coomassie staining to identify fractions containing the His_6_-tagged AKT1 proteins.

### 2.4. Size Exclusion Chromatography

Affinity purified AKT1 proteins variants were further purified by size exclusion chromatography using a 13 mL Superdex 200 (Cytiva Life Sciences) gel filtration column on the ÄKTA Pure L1 FPLC system. The column was equilibrated with 3 column volumes of buffer (20 mM HEPES pH 7.4, 300 mM NaCl, 5 mM β-mercaptoethanol). The pooled and concentrated fractions from the affinity chromatography purification were loaded onto the column with a flow rate of 0.5 mL/min and 1 mL elution fractions were collected. Elution fractions were separated by 10% SDS-PAGE to identify fractions containing purified proteins and pure fractions were pooled and concentrated using Vivaspin 6 spin-column (5 mL, 10 kDa molecular weight cutoff (MWCO), #14558502, Sartorius Canada, Oakville, ON, Canada). Concentrated AKT1 proteins were dialyzed into storage buffer (20 mM HEPES pH 7.4, 150 mM NaCl, 5 mM β-mercaptoethanol, 10% glycerol) and stored at −80 °C until further use.

### 2.5. Anion Exchange Chromatography

pAKT1^T308^ protein variants were further purified by anion exchange chromatography using the ÄKTA Pure L1 FPLC system (Cytiva Life Sciences) with a HiTrap Q HP anion exchange 1 mL column. The column was equilibrated with 5 column volumes of buffer A1 (20 mM HEPES pH 7.4, 150 mM NaCl, 5 mM β-mercaptoethanol). Unbound proteins were washed with 10 column volumes of buffer A1, and pAKT1 proteins were eluted with a gradient of 0 to 100% buffer B1 (20 mM HEPES pH 8.0, 300 mM NaCl, 5 mM β-mercaptoethanol). The flow rate was maintained at 0.5 mL/min and 1 mL fractions were collected. Fractions were separated by 10% SDS-PAGE and fractions containing pure pAKT protein were pooled and concentrated using a Vivaspin 20 concentrator (25 mL, 10 kDa MWCO, Sartorius, #14558502). Purified pAKT1 proteins were dialyzed into storage buffer (20 mM HEPES pH 7.4, 150 mM NaCl, 5 mM β-mercaptoethanol, 10% glycerol) and stored at −80 °C until further use.

### 2.6. AKT1 Kinase Activity Assay

To assure equal loading in all assays, purified AKT protein concentrations were measured using a Bradford assay (Biorad Canada, Mississauga, ON, Canada). The activity of each AKT1 and TAT-AKT1 variant was characterized by performing kinase assays in the presence of 200 µM substrate peptide CKRPRAASFAE (SignalChem, Vancouver, BC, Canada) derived from the natural AKT1 substrate GSK-3β. Kinase assays were performed in a buffer containing 25 mM 3-(N-morpholino)propanesulfonic acid (MOPS) pH 7.0, 12.5 mM β-glycerolphosphate, 25 mM MgCl_2_, 5 mM ethylene glycolbis(β-aminoethyl ether)-N,N,N′,N′-tetraacetic acid (EGTA) pH 8.0, 2 mM EDTA, 0.2 mM ATP, and 0.4 µCi (33 nM) [γ-^32^P]-ATP in a 30 µL reaction volume. Reactions were incubated at 37 °C with agitation and time points were taken over a 15 min time course. Reactions were initiated by the addition of 18 pmol of the specific AKT1 variant for a final enzyme concentration of 600 nM and reactions were quenched by spotting 3 µL aliquots of reaction solution on P81 paper at 5, 10, and 15 min. Following washes with 1% phosphoric acid (3 × 10 min) and 95% ethanol (1 × 5 min), the P81 paper was air-dried. Addition of radioactive ^32^P to the GSK-3β peptide was detected by phosphorimaging and imaged using a Storm 860 Molecular Imager (Molecular Dynamics, Sunnyvale, CA, USA). Spot intensity was quantitated using ImageQuant TL software (Cytiva Life Sciences).

### 2.7. Protein Incubation with Cells

HEK 293T cells were maintained in Dulbecco’s modified eagle’s medium (DMEM, Cellgro, ThermoFisher Scientific, Ottawa, ON, Canada) containing 10% fetal bovine serum and 1% penicillin/streptomycin at 37 °C with 5% CO_2_. Equal numbers of cells were seeded onto 6 well plates and cultured for 2–4 days until 90% confluence. AKT1 or TAT-AKT1 protein variants were added to a final concentration of 1 µM. After 1 h incubation, cells were washed twice with phosphate-buffered saline (PBS), harvested, and stored at −80 °C until further use.

### 2.8. Transfection of HEK 293T Cells 

A pcDNA3.1 plasmid encoding mCherry-AKT1 [33] was used to genetically over-express AKT1 in HEK 293T cells. The same number of cells were plated onto 6 well plates, and cells were transfected with 1 µg of pcDNA3.1 plasmid using Lipofectamine 3000 (ThermoFisher Scientific, #L3000015) according to the manufacturer’s instructions at 70% confluence. At 24 h after transfection, cells were stimulated with epidermal growth factor (EGF) for 10 min, harvested, and stored at −80 °C until further use.

### 2.9. Western Blotting 

For cells incubated with AKT1 or TAT-AKT1 protein variants or for cells transfected with pCDNA3.1-AKT1, protein extraction was initiated by suspending harvested cells in cold lysis buffer (50 mM Na_2_HPO_4_, 1 mM Na_4_P_2_O_7_, 20 mM NaF, 2 mM EDTA, 2 mM EGTA, 1 mM dithiothreitol, 300 µM PMSF, and protease inhibitor cocktail (Roche Canada, #04693159001)) and incubated on ice for 10 min with vortexing every 2 min. Lysates were cleared by centrifugation at 15,000× *g* for 15 min at 4 °C. Protein concentration was measured by Bradford assay and equal concentration of proteins (50 µg) were separated in 10% SDS-PAGE gel for each sample.

For western blotting of the purified recombinant AKT1 proteins, proteins were denatured using 3 × SDS sample loading buffer and extracted proteins from HEK 293T cells were diluted to separate 50 µg of protein by 10% SDS-PAGE. Proteins were transferred to a polyvinylidene fluoride (PVDF) membrane using a Turbo-Blot Turbo transfer system (Biorad Canada). The membranes were blocked with either 5% bovine serum albumin (BSA) in 1 × tris buffered saline with 0.1% Tween-20 (TBST) or 5% milk in 1 *×* PBS with 0.1% Tween-20 (PBST) for 1 h at room temperature. The membrane was immunoblotted with the indicated primary antibody overnight at 4 °C followed by 3 *×* 10 min washes in TBST or PBST. Next, the membrane was incubated with a secondary antibody for 1 h at room temperature and subsequently washed 3 *×* 10 min in TBST. Membranes were stored in TBS or PBS for further analysis. The blots were visualized by fluorescence on a LiCOR imaging system (LiCOR Biosciences, Lincoln, NE, USA). The following primary antibodies were used: AKT1, Cell Signaling Technology, Danvers, MA, USA #2938; pAKT (Thr308), Cell Signaling Technology #4060; GSK-3α/β, Santa Cruz Biotechnology sc-7291 (0011-A); pGSK-3α/β, Cell Signaling Technology #9331S; ribosomal protein S6, Cell Signaling Technology #2317S; and pS6 (S240/S244), Cell Signaling Technology #2215S. A glyceraldehyde 3-phosphate dehydrogenase (GAPDH) antibody (Abcam, Waltham, MA, USA, #ab8245) was used as loading control. The secondary fluorescent antibodies were Goat-anti-Mouse (Sigma-Aldrich Canada, #AQ127) and Donkey-anti-Rabbit (Sigma-Aldrich Canada, #AP182P).

### 2.10. Microscopy and Cell Imaging 

HEK 293T cells were cultured in 6 well dishes containing DMEM (Cellgro, ThermoFisher Scientific) supplemented with 10% fetal bovine serum and 1% penicillin/streptomycin at 37 °C with 5% CO_2_. For cell imaging experiments, cells were cultured in 24 well plates and 0.5 µM of each AKT1 protein variant was added to wells when cells reached 90% confluence and then incubated for 24 h. Following incubation with the AKT1 protein variants, cells were washed twice with DMEM prior to imaging. For HEK 293T cells incubated with mCherry-AKT1 protein variants or for cells transfected with plasmid encoded mCherry-AKT1, the cells were imaged at 24 h after incubation or transfection with an EVOS FL Auto 2 imaging system (ThermoFisher) using brightfield and fluorescence imaging. To detect mCherry fluorescence, cells incubated with TAT-mCherry-AKT1 protein variants or cells transfected with the mCherry-AKT1 plasmid were imaged with the RFP filter cube (excitation 542 ± 20 nm, emission 593 ± 40 nm). All images were captured with the EVOS 4× objective (fluorite, PH, long-working distance, 0.13 numerical aperture/10.58 mm working distance). Fluorescent images from wells representing three biological replicates for each condition were quantified using Image J software (National Institutes of Health, National Institute of Mental Health, Bethesda, MD, USA). Image J was used to count the total number of cells in brightfield images and then used to determine the total number of red fluorescing cells to calculate transfection efficiency. Statistical analysis was performed using GraphPad Prism software (GraphPad, San Diego, CA, USA). Details for confocal imaging are provided in the Supplementary Materials (S.3 Supplementary Methods).

### 2.11. Cytotoxicity Assay 

Equal numbers of HEK 293T cells were seeded in 96 well dishes containing DMEM (Cellgro, ThermoFisher Scientific) supplemented with 10% fetal bovine serum and 1% penicillin/streptomycin at 37 °C with 5% CO_2_. At 90% confluence, 0.5 µM of TAT-AKT1 protein variants were added to each well and incubated for 24 h. The CytoTox-Glo cytotoxicity assay kit (Promega, #G9290, Madison, WI, USA) was used to measure the total cell and dead cell numbers by following the supplied protocol. Each experiment was performed in three biological replicates.

### 2.12. Trypan Blue and Sytox Blue Assays 

Equal numbers of HEK 293T cells were cultured in 96 well plates containing DMEM (Cellgro) supplemented with 10% fetal bovine serum and 1% penicillin/streptomycin at 37 °C in 5% CO_2_. When cell confluence reached 90%, TAT-AKT1 and TAT-pAKT1^T308^ proteins were added at a concentration of 1 µM and incubated for 1 h or 24 h. An aliquot of 100 µL of cells from each well was transferred to a microcentrifuge tube and 100 µL of trypan blue dye was added. Viable and dead cells were counted under a light microscope after 1 h or 24 h of protein delivery. Sytox blue (ThermoFisher, #S34857) was used to measure the fluorescence of dead cells [36] at 24 h after TAT-AKT1 protein incubation. Cell images were taken with a EVOS FL Auto 2 cell imaging system using a CFP filter cube (emission 445 ± 45 nm, excitation 510 ± 42 nm). Dead cell fluorescence was quantified using image J software. Each experiment was performed in three biological replicates.

### 2.13. Quantification and Statistical Analysis 

For all experiments, at least three biological replicates were used and indicated on all graphs. Graphs were generated in Microsoft Excel. Western blots were quantified using Image Lab (Biorad) and the data were compiled and normalized in Microsoft Excel. *p*-values were calculated by one-way analysis of variance (ANOVA), two-way ANOVA, or Student’s two-tailed *t*-test as indicated.

## 3. Results

### 3.1. Production and Purification of Recombinant AKT1 and Site-Specifically Phosphorylated AKT1 Variants

TAT peptide tags were shown by us [37] and others [38,39,40] to enable rapid and efficiency delivery of recombinant proteins to mammalian cells. To determine if a TAT-peptide fusion will enable delivery of recombinant AKT1 to human cells (Figure 1B), we first constructed, produced, and purified AKT1 variants with a TAT tag as well as AKT1 variants lacking the TAT-tag as before [19,33,34]. Full-length human AKT1 variants were expressed and produced in *E. coli* to generate unphosphorylated AKT1 or TAT-tagged AKT1. We also produced pAKT1^T308^ variants with or without the TAT-tag by co-expressing each variant with PDK1 to produce site-specific phosphorylated pAKT1^T308^ and TAT-pAKT1^T308^. Using a similar approach to that we established previously [33], all variants were initially purified using Ni^2+^-NTA affinity chromatography relying on the His_6_-tag included in each construct (see Supplementary Material Section S.2). The unphosphorylated variants were purified further by size exclusion chromatography leading to pure fractions (Figure S1A,C). Adhering to our previous approach [33], the phosphorylated variants were purified by size exclusion and then further purified by anion exchange chromatography to yield pure fractions of pAKT1^T308^ (Figure S1B) and TAT-pAKT1^T308^ (Figure S1D).

To confirm incorporation of pThr308 in both the wild-type and TAT-tagged AKT1 variants we performed western blotting on the purified proteins (Figure S2). We blotted the purified proteins with both an AKT1 antibody (Figure S2A) and an antibody specific for pAKT^T308^ (Figure S2B). The AKT1 antibody confirmed equivalent loading of each of the purified AKT1 and TAT-AKT1 variants, and we found that the pAKT^T308^ specific antibody displayed a robust response to only the pAKT1^T308^ and TAT-pAKT1^T308^ variants. The blots indicate an equivalent level of phosphorylation on the pAKT1^T308^ and TAT-pAKT1^T308^ proteins (Figure S2C). We previously used multiple mass spectrometry approaches to characterize the pAKT1^T308^ variant and found stoichiometric incorporation of phosphate at the Thr308 site [19,33,34]. Here, we performed liquid chromatography tandem mass spectrometry (LC-MS/MS) analysis of the TAT-pAKT1^T308^ protein and independently confirmed incorporation of pThr308 (Figure S2D). In agreement with our previous studies of pAKT1^T308^ [33], the TAT-pAKT1^T308^ protein showed no evidence of phosphorylation at the other regulatory site, Ser473. Indeed, we observed only the Ser473 peptide (Figure S2E).

### 3.2. Enzymatic Activity of AKT1 and TAT-AKT1 Variants

**Figure 2:**
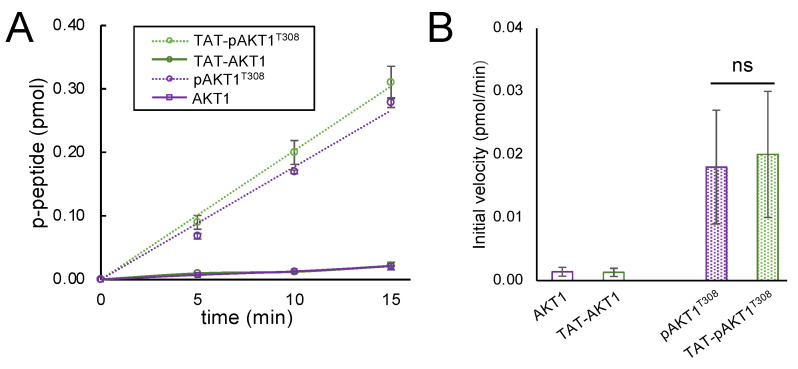
Enzymatic activity of AKT1 is dependent on phosphorylation at Thr308 and was not affected by fusion of AKT1 to the cell penetrating TAT-tag. (**A**) Time courses and (**B**) initial velocity of three independent in vitro kinase activity assays. AKT1 variants were incubated with a GSK-3β substrate peptide and [γ-^32^P]-ATP and spotted on filter paper. Unreacted [γ-^32^P]-ATP was washed away and reaction products visualized by phosphorimaging (Figure S3) and quantified. AKT1 (purple) and TAT-AKT1 (green) did not show significant activity above background, whereas pAKT1^T308^ (purple dotted) and TAT-pAKT1^T308^ (green dotted) were enzymatically active. The TAT-tag did not significantly alter pAKT1^T308^ activity. Significant differences were calculated by two-tailed *t*-test (ns—not significant).

To determine if addition of the TAT-tag affected AKT1 activity, we tested the enzymatic activity of AKT1 variants with and without a TAT-tag. The activity of each AKT1 variant was investigated using a standard kinase assay, as before [19,33,34]. Briefly, we monitored the transfer of a radiolabeled γ-phosphate from [γ-^32^P]-ATP to a substrate peptide based on the natural AKT1 substrate, GSK-3β. We measured phospho-peptide production over a 15 min time course. While both the unphosphorylated AKT1 and TAT-AKT1 proteins showed no activity that was distinguishable from background measurements, we found that both pAKT1^T308^ and TAT-pAKT1^T308^ showed robust kinase activity that increased linearly during the time course (Figure 2A and Figure S3). There was no significant difference in the phosphorylation rate catalyzed by pAKT1^T308^ or TAT-pAKT1^T308^ (Figure 2B and Figure S3), indicating that the TAT-tag does not perturb the activity of the enzyme. The activity data agree with our finding of a similar level of Thr308 phosphorylation in both the pAKT1^T308^ and TAT-pAKT1^T308^ variants (Figure S2B,C).

### 3.3. Delivery of TAT-AKT1 Variants to Human Cells

We used the HEK 293T cell line as a model system to characterize TAT-tag dependent delivery of AKT1 to human cells. In future and on-going studies beyond the scope of this work, we will also deliver TAT-AKT1 variants to other mammalian cell types. HEK 293T cells are well established cell biological model systems that display robust growth and high transfection efficiencies [41]. This aspect was important for our comparison of TAT-dependent AKT1 delivery to more traditional transient transfection approaches described below (see Section 3.6). AKT1 is also highly expressed in many human tissues, including the kidney [42], making HEK 293T cells an appropriate model system to study AKT1 signaling. Indeed, some of the initial characterizations of AKT1 activity [43] as well as more recent studies to screen AKT1 inhibitors [44] and to define new roles for AKT1 in diverse cellular processes including RNA metabolism [45,46], miRNA regulation [31], and non-sense mediated decay [47] each relied on HEK 293 cells as an appropriate cell line to investigate AKT1 biology.

**Figure 3:**
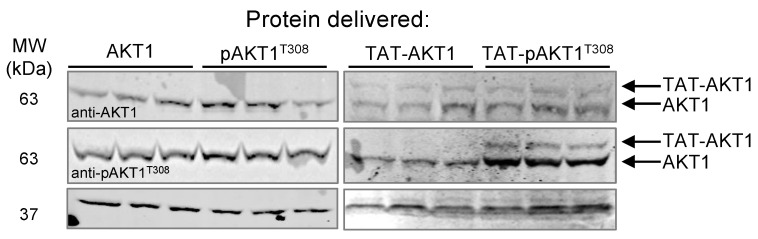
Delivery of TAT-AKT1 protein variants to HEK 293T cells. HEK 293T cells were incubated with AKT1, pAKT1^T308^, TAT-AKT1, or TAT-pAKT1^T308^ for 1 h in three biological replicates. Cell extracts were separated by SDS-PAGE and immunoblotted with AKT1, pAKT^T308^, and GAPDH specific antibodies. Cells incubated with AKT1 or pAKT1^T308^ showed a single band in all blots corresponding to endogenous AKT1. Two bands, corresponding to endogenous AKT1 and TAT-AKT1 separated by size, were apparent in cells transfected with TAT-tagged AKT1 variants, demonstrating effective delivery of both TAT-tagged AKT1 variants into cells. TAT-AKT1 delivery did not lead to phosphorylation of TAT-AKT1 in cells, as no phosphorylated TAT-AKT1 was detected by the pThr308 specific antibody after 1 h. The delivered TAT-pAKT1^T308^ was readily detected by phospho-specific antibody for pAKT^T308^.

Cells were grown to 90% confluence and then incubated with one of the AKT1 variants characterized above. After a 1-h incubation of the cells with AKT1, pAKT1^T308^, TAT-AKT1, or TAT-pAKT1^T308^, we used western blotting to demonstrate the TAT-tag mediated cellular uptake and to monitor the phosphorylation status of endogenous AKT, TAT-AKT1 and TAT-pAKT1^T308^ using specific antibodies for AKT1, pAKT1^T308^, and GAPDH as a loading control. The AKT1 and pAKT1^T308^ constructs contain a His_6_-tag derived from pCDF-Duet-1 as described before [33] that have a relative molecular weight of 3.1 kDa greater than the endogenous AKT1. The TAT-tagged AKT1 variants have a larger N-terminal tag, including both His_6_ and the TAT peptide, derived from the pTAT-HA plasmid [35] (see Supplementary Material Section S.2). Thus, the TAT-tagged AKT1 variants were easily differentiated from endogenous AKT1 in the western blots due to their size difference of 6 kDa (Figure 3).

Following the protein incubation period, the cells were extensively washed with fresh media to remove any protein remaining outside of the cells. In western blots, we clearly observed a band in the anti-AKT1 blots corresponding to the higher molecular weight of the TAT-AKT1 proteins relative to endogenous AKT (Figure 3). In contrast, in cells incubated with the AKT1 variants lacking the TAT-tag, we observed only the endogenous AKT1 and no additional bands corresponding to the His_6_-AKT1 or His_6_-pAKT1^T308^ proteins. In agreement with our characterization of the purified TAT-AKT1 and TAT-pAKT1^T308^ proteins (Figure S3), only the TAT-pAKT1^T308^ protein was detected by the pAKT^T308^ specific antibody (Figure 3). Together the data show that both the TAT-AKT1 and TAT-pAKT1^T308^ proteins were delivered to the interior of the cells, and further, that the TAT-pAKT1^T308^ retained phosphorylation at Thr308 while the TAT-AKT1 protein did not acquire Thr308 phosphorylation in the cells (Figure 3).

To further verify that the AKT1 proteins lacking the TAT-tag were not taken up by the cells, we performed an independent experiment where HEK 293T cells were incubated with buffer only, with AKT1, or with pAKT1 proteins lacking the TAT-tag, and we found no change in the level of AKT1 compared to a GAPDH loading control in any of these conditions (Figure S4). Thus, the TAT-tag was necessary and sufficient to delivery TAT-AKT1 and TAT-pAKT1^T308^ variants to human cells.

### 3.4. Selective Phosphorylation of GSK-3α by TAT-pAKT1^T308^

GSK-3 was the first identified AKT1-dependent substrate [48]. GSK-3 has two paralogs that are often referred to as isoforms, including a higher molecular weight GSK-3α and a lower molecular weight GSK-3β protein [49]. A typical approach to promote AKT activity in cells involves genetic expression of a myristoylated-AKT1 variant (myr-AKT1) and subsequent stimulation of cells with insulin or growth factors such as EGF [50] or platelet derived growth factor (PGDF) [51]. The myr-tag anchors AKT1 to the plasma membrane. Studies with PGDF show myr-AKT1 is activated and causes increased phosphorylation of GSK-3α at Ser21 (SGRARTSsFAEPGGG) and GSK-3β at Ser9 (SGRPRTTsFAESCKP) [51]. The AKT1 target peptides in GSK-3α and GSK-3β are homologous but not identical, and both can be visualized using anti-GSK-3 and anti-pGSK-3 specific antibodies.

**Figure 4:**
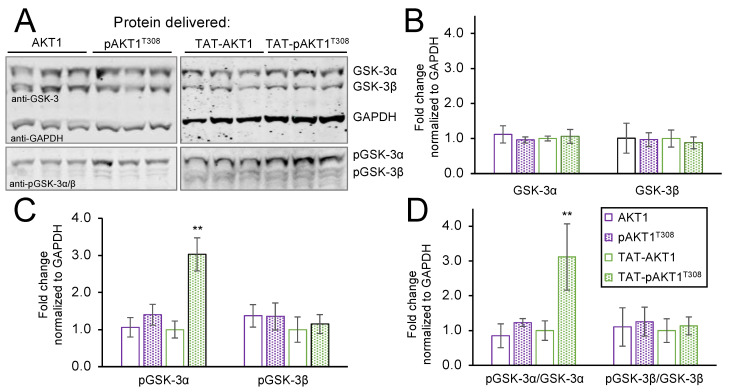
TAT-pAKT1^T308^ protein delivery stimulates AKT1 signaling. HEK 293T cells were incubated with AKT1, pAKT1^T308^, TAT-AKT1, or TAT-pAKT1^T308^ for 1 h in three biological replicates. (**A**) Cell extracts were separated by SDS-PAGE and immunoblotted with GSK-3, pGSK-3, and GADPH specific antibodies. GSK-3 homologs GSK-3α and GSK-3β are direct substrates of AKT1. (**B**) Quantification of western blots showed no change in GSK-3α or GSK-3β protein abundance. Changes in phosphorylation of GSK-3α or GSK-3β were quantified and normalized to (**C**) GAPDH or (**D**) GSK-3α and GSK-3β, respectively, showing that TAT-pAKT1^T308^ specifically stimulates phosphorylation of GSK-3α, but not GSK-3β. Significant differences were calculated by two-tailed *t*-test and are indicated by asterisks (** *p* < 0.01).

To determine if TAT-pAKT1^T308^ is active once delivered to cells, we used western blotting to probe cells for GSK-3 and pGSK-3 levels following incubation of cells with AKT1, pAKT1^T308^, TAT-AKT1, or TAT-pAKT1^T308^ proteins (Figure 4). Following incubation of HEK 293T cells with each of the indicated AKT1 variants, we identified no significant changes in the levels of GSK-3α or GSK-3β in comparison to a GAPDH loading control (Figure 4A,B). We next compared the levels of pGSK-3α and pGSK-3β to the GAPDH loading control (Figure 4A,C), and we also determined the ratio of phosphorylated GSK-3α and GSK-3β to the levels of the respective un-phosphorylated proteins (Figure 4D). In agreement with our findings above, neither of the AKT1 variants lacking the TAT-tag led to any stimulation of GSK-3 phosphorylation. In addition, the unphosphorylated TAT-AKT1 protein did not alter GSK-3 phosphorylation levels as anticipated. Strikingly, TAT-pAKT1^T308^ protein delivered to cells showed a robust 3-fold and significant increase in GSK-3α phosphorylation but no stimulation of GSK-3β phosphorylation was detected (Figure 4C,D). The data demonstrate that increased pGSK-3α levels were dependent on both the TAT-tag and the phosphorylation status of the delivered AKT1 protein.

### 3.5. TAT-pAKT1^T308^ Stimulates Downstream AKT1 Signaling to Ribosomal Protein S6

**Figure 5:**
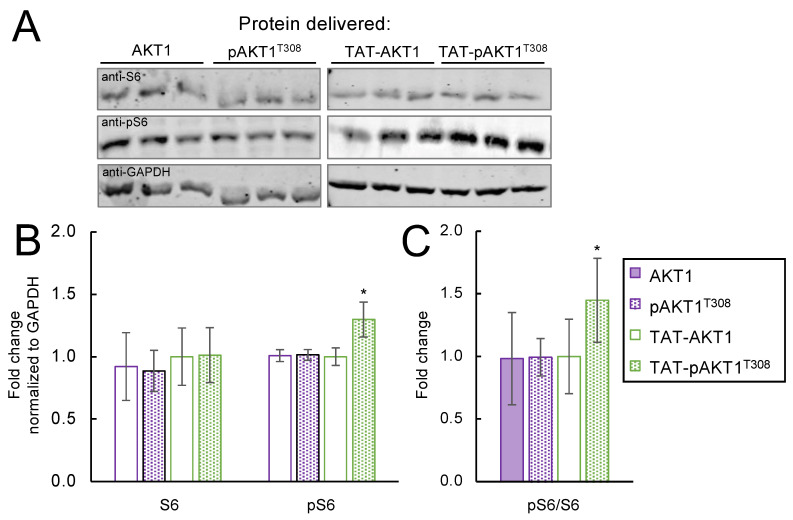
TAT-pAKT1^T308^ protein delivery stimulates downstream AKT1 signaling to ribosomal protein S6. HEK 293T cells were incubated with AKT1, pAKT1^T308^, TAT-AKT1, and TAT-pAKT1^T308^ for 1 h in three biological replicates. (**A**) Western blots and (**B**,**C**) quantification of blots of cell extracts separated by SDS-PAGE and immunoblotted with ribosomal protein S6, pS6, and GADPH specific antibodies. S6 phosphorylation was significantly increased after incubation with TAT-pAKT1^T308^, but not following incubation of cells with AKT1 or pAKT1 lacking the TAT tag or with unphosphorylated TAT-AKT protein variants. Significant differences were calculated by two-tailed *t*-test and are indicated by asterisks (* *p* < 0.05).

Although AKT1 is thought to regulate many cellular substrates directly and signaling pathways indirectly, one of the best characterized downstream pathways involves AKT1-dependent stimulation of protein synthesis via indirect phosphorylation of the ribosomal protein S6 at Ser240/Ser244 (reviewed in [52]). AKT1 directly phosphorylates the mammalian target of rapamycin (mTOR), which in turn phosphorylates and activates p70 S6 kinase (p70S6K) that then targets and phosphorylates ribosomal protein S6 at Ser240/Ser244 [53,54]. To determine if TAT-pAKT1^T308^ stimulates a downstream signaling pathway of AKT1, we used western blotting to measure S6 and pS6 levels in cells incubated with AKT1, pAKT1^T308^, TAT-AKT1, or TAT-pAKT1^T308^ proteins (Figure 5A). We monitored S6 and pS6 levels relative to the GAPDH loading control (Figure 5A,B), and we determined the level of pS6 compared to the level of S6 protein in each condition (Figure 5A,C). We observed no significant changes in S6 protein levels in each condition (Figure 5B), and we found no significant changes in pS6 levels in cells treated with AKT1, pAKT1^T308^, or TAT-AKT1. We did observe a significant 1.5-fold stimulation of S6 phosphorylation at Ser240/Ser244 only in cells treated TAT-pAKT1^T308^ (Figure 5B,C). The data indicate that TAT-pAKT1^T308^ stimulates signaling pathways downstream of AKT1.

### 3.6. Genetic Over-Expression Model of AKT1 Activity in Stimulated Cells

**Figure 6:**
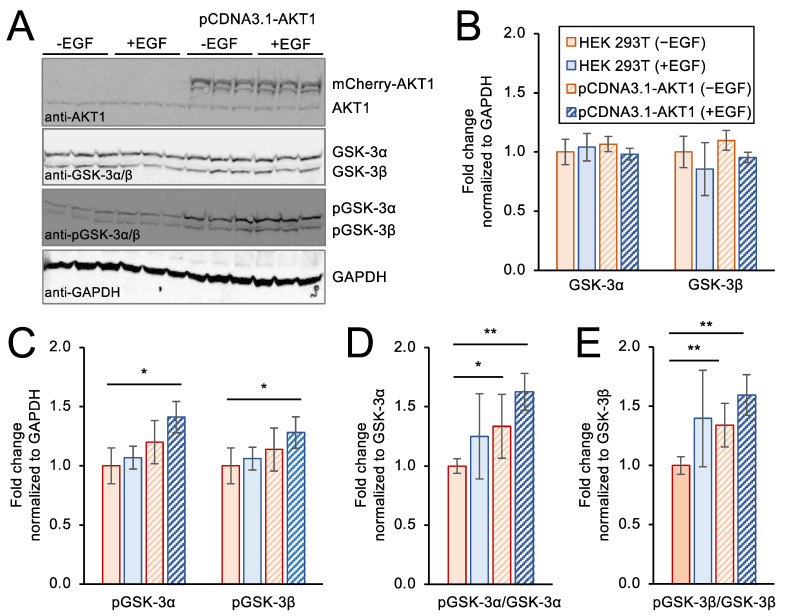
AKT1 over-expression and growth factor stimulation activates AKT1 signaling in HEK 293T cells. HEK 293T cells were transfected with an mCherry-AKT1 over-expression plasmid. At 24 h after transfection, cells were stimulated with EGF for 10 min. (**A**) Cell extracts were separated by SDS-PAGE and immunoblotted with AKT1, GSK-3, pGSK-3, and GAPDH specific antibodies in three biological replicates. (**B**) Quantification of western blots showed no change in GSK-3α or GSK-3β protein abundance. (**C**) GSK-3α and GSK-3β phosphorylation normalized to GAPDH was significantly increased in cells over-expressing AKT1 after EGF stimulation, but not significantly changed in EGF stimulated cells alone or unstimulated cells over-expressing AKT1. (**D**) GSK-3α and (**E**) GSK-3β phosphorylation was also normalized to GSK-3α and GSK-3β levels, showing increased phosphorylation of both GSK-3 isoforms in response to AKT1 over-expression, which was further increased upon EGF stimulation. Error bars represent the standard deviation of the mean. *p*-values were calculated by two-tailed *t*-test and are indicated by asterisks (* *p* < 0.05, ** *p* < 0.01).

As noted above, traditional approaches to generate cells with active AKT1 often involve addition of EGF to induce AKT1 phosphorylation and stimulate AKT signaling pathways [55]. To provide a direct comparison of this approach to our novel method to deliver active AKT1 to cells, we conducted a set of independent experiments to over-express AKT1 from a plasmid transiently transfected to HEK 293T cells (Figure 6). We used a well-established over-expression model of AKT1, in which an mCherry-tagged AKT1 protein is produced from a pCDNA3.1 backbone vector in the cells [33,55]. We used western blotting to show the level of endogenous AKT1 as well as to demonstrate expression of the plasmid encoded mCherry-AKT1 (Figure 6A). The data confirm over-expression of mCherry-AKT1 in cells transfected with the relevant plasmid and show a similar level of mCherry-AKT1 to the level of endogenous AKT1 observed in the same cells. We note that the plasmid-based mCherry-AKT1 expression leads to a similar level of additional AKT1 in the cells compared to our experiments with delivered TAT-tagged AKT1 variants (Figure 3).

We used western blotting to monitor the levels of GSK-3α and GSK-3β as well as pGSK-3α and pGSK-3β in untreated cells and in transfected cells with or without addition of EGF. None of the conditions caused any significant change in GSK-3α or GSK-3β levels relative to a GAPDH control (Figure 6A,B). As above, we monitored pGSK-3α and pGSK-3β levels relative to the GAPDH control (Figure 6C) as well as the levels of pGSK-3α and pGSK-3β compared to the levels of un-phosphorylated GSK-3α and GSK-3β, respectively (Figure 6D,E). Both approaches led to a consistent observation of a significant and ~1.5-fold increase in pGSK-3α and pGSK-3β levels in cells over-expressing AKT1 and stimulated with EGF relative to unstimulated control cells lines lacking AKT1 over-expression. These findings agree with previously published studies [50,51] where growth factor stimulation of cells with over-expressed AKT1 causes an increase in phosphorylation of both GSK-3α and GSK-3β.

### 3.7. Delivery Efficiency and Localization of TAT-Tagged AKT1

**Figure 7:**
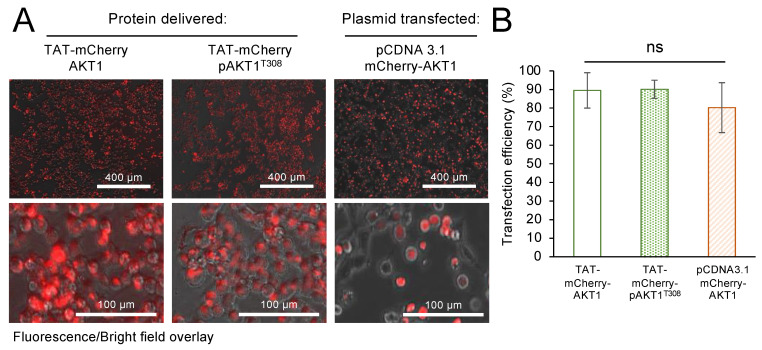
Delivery of TAT-mCherry-AKT1 variants compared to plasmid-based transfection efficiency of mCherry-AKT1. (**A**) Overlay of brightfield and fluorescent (excitation 531 nm, emission 593 nm) images (above) and zoomed-in images (below) of cells 24 h after incubation with TAT-mCherry-AKT1, TAT-mCherry-pAKT1^T308^, or lipofectamine mediated transfection with an mCherry-AKT1 expressing plasmid. (**B**) Quantification of delivery or transfection efficiency from three biological replicates was calculated as the ratio of transfected cells/total cells and by determining fluorescence of the mCherry-AKT1 fusion proteins in cells. Protein delivery and plasmid transfection efficiency was not significantly different. The data are based on three biological replicates, error bars show ±1 standard deviation, and significance was calculated by one-way ANOVA (ns—not significant).

To measure the efficiency of TAT-tagged AKT1 delivery to live human cells, we constructed, produced, and purified AKT1 and pAKT1^T308^ variants in *E. coli* that contain the TAT-tag followed by an N-terminal mCherry tag (see Supplementary Material Section S.2). Since traditional approaches to study AKT1 signaling in cells often rely on over-expression of mCherry-tagged AKT1 in transiently transfected cells [33,55,56,57], we determined the transfection efficiency of plasmid-borne mCherry-AKT1 compared to delivery of the TAT-mCherry-tagged AKT1 variants. At 24 h after transfection with pCDNA3.1 encoded mCherry-AKT1 or 24 h after incubation of cells with TAT-mCherry-AKT1 variants, the cells were imaged by fluorescence and brightfield microscopy. Based on the images, delivery efficiency was calculated for the TAT-mCherry-tagged proteins and plasmid-based transfection efficiency was also determined (Figure 7). In HEK 293T cells, we found that both the plasmid transfection and the TAT-tagged protein delivery were similarly efficient. The TAT-mCherry-AKT1 entered the cells with ~90% delivery efficiency, while in our plasmid-based transfection we recorded a mean of ~80% transfection efficiency. Statistical analysis showed that TAT-mCherry tagged AKT1 protein delivery was not significantly different from the plasmid-based transfection efficiency. Thus, the TAT-tagged protein transfection is similarly efficient compared to traditional approaches. In the case of the plasmid-based experiment, lipofectamine transfection reagent was employed, while the TAT-tagged proteins required no transfection or other additional reagents to induce efficient cellular uptake.

To observe the localization of TAT-AKT1, we included zoomed-in images of cells incubated with the TAT-mCherry-AKT1 variants as well as the plasmid-transfected cells. These images show that the TAT-tagged AKT1 variants and the plasmid expressed AKT1 are well-distributed in the interior of the cells (Figure 7). To get a more definitive image of TAT-AKT1 delivered to cells, we also conducted confocal fluorescence microscopy (see Supplementary Material Section S.3) of HEK 293T cells incubated with TAT-AKT1 (Figure S5A,B) or TAT-pAKT1^T308^ (Figure S5C,D) variants. The TAT-AKT1 proteins were visualized with an anti-TAT antibody and fluorescent secondary antibody, while the nuclei were stained with 4′,6-diamidino-2-phenylindole (DAPI) and the membranes were stained with an AlexaFluor-linked phalloidin (Figure S5). The confocal images agree with our TAT-mCherry-AKT1 studies and demonstrate that the TAT-AKT1 variants are well-distributed in the cytoplasm.

### 3.8. Cellular Viability and Toxicity

To quantify the impact on cell viability following protein delivery of TAT-AKT1 protein variants, we conducted trypan blue assays on cells incubated with TAT-AKT1 or TAT-pAKT1^T308^ protein for 1 h (Figure S6A) or 24 h (Figure S6B). In the trypan blue assay, dead and live cells were counted using brightfield imaging. Cells treated with TAT-AKT1 or TAT-pAKT1^T308^ showed >94% cell survival compared to the control cells incubated with buffer only (Figure S6A,B).

**Figure 8:**
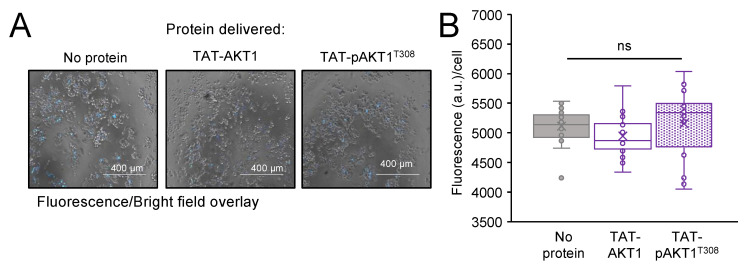
TAT-AKT1 protein delivery is non-toxic to HEK 293T cells. Cytotoxicity was measured after a 24-h incubation with no protein (buffer only), TAT-AKT1, or TAT-pAKT1^T308^ by staining dead cells with Sytox blue in three biological replicates. (**A**) Images of cells with Sytox blue staining of dead cells. (**B**) Quantification of dead cells shows no significant impact of TAT-AKT1 and TAT-pAKT1^T308^ protein delivery compared to cells treated with buffer only (no protein). Significance was calculated by one-way ANOVA (ns—not significant).

Next, dead cell nuclei were stained with the fluorescent Sytox blue probe and images were taken by fluorescence microscopy at 24 h after incubation with no protein (buffer only), with TAT-AKT1, or with TAT-pAKT1^T308^ (Figure 8). There was no significant difference in the fluorescence from dead cells in comparing cells transfected with either TAT-AKT1 variant or without protein.

Finally, a cytotoxicity assay was conducted to measure potential toxicity associated with either of the TAT-tagged AKT1 variants. The same number of HEK 293T cells were seeded into the 96 well plates in culture media. Following a 24-h incubation of the cells with buffer only (no protein), with TAT-AKT1, or with TAT-pAKT1^T308^ proteins, the live-cell impermeant peptide substrate aminoluciferin (AAF-Glo) was added into each well to assess the percentage of dead cells in a microplate reader. Following cell lysis, the luminescent signal was used to count the total number of cells. Cells transfected with no protein (buffer only), TAT-AKT1, or TAT-pAKT1^T308^ each showed no significant difference in cytotoxicity at 24 h after protein incubation (Figure S6C). Together the data show that TAT-tagged AKT1 variants are not toxic to cells, nor do they reduce cell viability.

## 4. Discussion

### 4.1. Fusing AKT1 with the Cell Penetrating Peptide TAT

We engineered AKT1 and pAKT1 variants to include an N-terminal TAT tag to facilitate their cellular uptake into mammalian cells. The TAT peptide is a member of a diverse and growing collection of cell penetrating peptides (CPPs) that have been used to deliver small molecules [58], proteins [39], and even mRNAs [59] to mammalian cells. The TAT peptide (YGRKKRRQRRR) is a fragment from a larger TAT protein that is essential for HIV replication in cells [60]. Early studies identified a region of the TAT protein (residues 37–72) that is responsible for cellular uptake [38]. That same segment of TAT was also fused to a variety of other proteins, such as β-galactosidase and horseradish peroxidase, to facilitate their uptake into mammalian cells [40]. Recently, we fused the TAT-tag to the human selenocysteine-containing thioredoxin reductase 1, and demonstrated that the TAT-tag was necessary and sufficient to enable cytosolic delivery of an active selenoprotein to human cells for the first time [37].

Previous studies have appended His-tags [33], mCherry [55], and glutathione tags [61] to the N-terminus of AKT1, and each study found that the N-terminally tagged AKT1 retained kinase activity. Because of the important role of the C-terminal HM domain in AKT1 activation [15], C-terminal tagging is not appropriate for AKT1. Since TAT-fusions of AKT1 had not been created before, we characterized the biochemical activity of AKT1 and pAKT1^T308^ with and without the TAT-tag. We confirmed that the addition of a TAT-tag at the N-terminus does not alter catalytic activity of the AKT1 protein variants using in vitro kinase assays with a substrate peptide derived from GSK-3β.

### 4.2. Impact of TAT-Tagged Protein Delivery on Cell Fitness

One report suggested that TAT-tag molecules, such as TAT-NEMO binding domain (NBD), can have a cytotoxic effect at high concentration in treated cells. Incubation with a TAT-tagged NBD peptide led to decreased cell numbers at concentration of 100 µM [62]. We found that following incubation with human cells in culture, TAT-AKT1 and TAT-pAKT1^T308^ proteins at concentrations of 0.5 to 1 µM provided a level of delivered TAT-AKT1 that was similar to the level of endogenous AKT1 and that did not cause significant changes in cell viability or cytotoxicity according to multiple independent assays. More than 94% cells were viable following incubation with no protein or with either TAT-tagged AKT1 variant. As the cell survival rate was similar to the untreated cells, we measured cytotoxicity in cells harboring TAT-tagged AKT1 variants. We used a sensitive luminescent cytotoxicity assay to determine the relative number of dead cells in which the intensity of luminescence directly associates with the number of cells undergoing cytotoxic stress [63,64]. Compared to untreated HEK 293T cells, cells incubated with TAT-AKT1 variants did not show significant increases in dead cell numbers or in cells undergoing cytotoxic stress. The data underline the effectiveness of the cell penetrating TAT-tag for rapid and efficient delivery of active or inactive recombinant human AKT1 through the cell membrane without significantly compromising cellular viability or toxicity.

### 4.3. Delivery of Active TAT-pAKT1^T308^ Stimulates AKT1 Signaling in Human Cells

We utilized the TAT-mCherry-AKT1 to visualize protein delivery and calculate delivery efficiency. Independently, we utilized the TAT-pAKT1 variants lacking the mCherry fusion protein to investigate AKT1-dependent signaling. TAT-tagged protein delivery efficiency was ~90% after 24 h demonstrating efficient protein uptake by the cells. In comparison, transfection of cells with a plasmid bearing mCherry-AKT1 showed a similar (~80%) and not significantly different level of transfection efficiency. Together the data suggest that TAT-tagged AKT1 delivery is of similar efficiency to traditional plasmid-based approaches in HEK 293T cells, however, the TAT-tagged protein was delivered to cells without additional transfection reagents and can be applied to any recombinantly produced protein, including proteins with non-canonical amino acids [37].

AKT-mediated downstream regulation of effector proteins is directly linked to glycogenosis, glucose import, and protein synthesis [65,66,67]. GSK-3 is a highly conserved regulatory enzyme, and inhibition of GSK-3 activity by AKT-dependent phosphorylation was implicated in cancer progression, neuronal disease, aging, and metabolic disorders such as diabetes [66]. The GSK-3α and 3β isoforms have distinct regulatory roles and they do not compensate for each other’s functions. GSK-3 is a critical downstream target of the PI3K-AKT cell signaling pathway, and GSK-3 activity is controlled in insulin or growth factor stimulated cells by AKT-dependent phosphorylation of GSK-3α at Ser21 and of GSK-3β at Ser9 [48]. The substrate specificity and recognition capabilities of GSK-3α and 3β also vary between and within tissues. In brain cells, deletion of each GSK-3 isoform produces distinct substrate phosphorylation of Collapsin response mediator protein (CRMP). CRMP Thr509, Thr514, and Ser518 phosphorylation is observed in GSK-3α depleted cells, but not GSK-3β depleted cells [68]. A knockdown of GSK-3α in mice showed increased sensitivity towards insulin, suggesting the significant role of GSK-3α in glucose synthesis and as a therapeutic target for diabetes [69]. Therefore, GSK-3 isoforms show a clear variation in their functions, and the distinct roles of GSK-3α and GSK-3β need to be delineated to understand the mechanism underlying various disorders.

In cells that we transfected with an AKT1 over-expression construct and then stimulated with growth factor, we reproduced the well-established result that AKT1 caused increased phosphorylation of its target substrates on GSK-3α and GSK-3β. In contrast, cells incubated with TAT-pAKT1^T308^ showed a strong and selective induction of phosphorylation on GSK-3α only. We observed evidence that TAT-pAKT1^T308^ stimulates direct downstream AKT1 substrates, such as GSK-3α, and we also observed evidence of downstream stimulation of ribosomal protein S6 phosphorylation by TAT-pAKT1^T308^ likely due to the well-established role of AKT1 in activation of the mTOR pathway [22,23,24].

We showed before that peptides representing the GSK-3α and GSK-3β (Figure 2) AKT1 phosphorylation sites are both competent substrates for pAKT1^T308^ [19]. We previously observed that pAKT1^T308^ is significantly more active with a GSK-3α peptide compared to a doubly phosphorylated ppAKT1^T308,S473^ [19], while pAKT1^T308^ and ppAKT1^T308,S473^ showed similar and robust activity with a GSK-3β peptide [19]. Taken together with the data presented here, our observations suggest that either doubly phosphorylated ppAKT1^T308,S473^ or growth factor stimulation of other kinases are responsible for phosphorylation of GSK-3β in cells. In the context of cells, scaffolding effects [70] and the role of phosphatases [71] are important factors for the accumulation of phosphorylation on specific sites that may also explain the apparent selective activity of TAT-pAKT1^T308^ on GSK-3α in cells. Fascinatingly, the phosphatase laforin is selective for de-phosphorylation of GSK-3β at Ser9 and has no effect on GSK-3α phosphorylation at Ser21 [72]. Insulin stimulation represses laforin protein levels [73] and enhances GSK-3β pSer9, while the same site is hyper-phosphorylated in laforin knock-out mice [73]. Over-expression of laforin is sufficient to abrogate GSK-3β phosphorylation even in insulin stimulated HEK 293 cells [74]. It is, therefore, feasible that TAT-pAKT1^T308^ phosphorylates both GSK-3α and GSK-3β, and GSK-3β is subsequently dephosphorylated by laforin. We will test this hypothesis in future work.

### 4.4. TAT-Fusion of Peptides and Proteins to Modulate AKT1 Signaling

The TAT-peptide was used in previous studies of AKT1, but no other studies fused TAT to the full-length or to the activated AKT1 as we have demonstrated here. Previously, the TAT-peptide was fused to the PH domain of AKT1 (residues 1 to 147) to generate a dominant negative and truncated version of AKT1 lacking the kinase domain [75]. The TAT-PH protein was successfully delivered to cells and was found to inhibit the anti-apoptotic effects of estrogen in vascular endothelial cells [75]. TAT was also fused to other peptides, such as a segment of the angiotensin II type I receptor to partially inhibit AKT signaling in HEK 293 cells [76], or to a fragment of the NF-κB essential modulator-binding domain to generate a 33 amino acid peptide that was shown to reduce expression of AKT [77]. TAT-peptides were also conjugated to nanoparticles in approaches to improve delivery of chemotherapeutics that disrupt AKT1 signaling [78,79]. There are many examples in the literature of fusing TAT to peptides or other compounds that inhibit human kinases, including inhibitors of the EGF receptor [80], protein kinase C [80], and casein kinase 2 [81].

Interestingly, a larger version of the TAT-peptide (residues 48–60) itself was shown to inhibit AGC family kinases [82]. The TAT-peptide (residues 48–60, GRKKRRQRRRAHQ) was found to be a µM level inhibitor of protein kinase A. The report also showed that a concentration of 10 µM of the TAT_48–60_ peptide was required to have a substantial impact on AKT kinase activity, and a concentration of 30 µM TAT_48–60_ peptide was needed to reduce ERK2 phosphorylation in HeLa cells. These levels are 30 to 60-fold above the concentrations in which we were working, and we used a TAT-peptide fused to AKT rather than a TAT-peptide in isolation. Furthermore, our TAT-fusion peptide is composed of residues 47–57 (YGRKKRRQRRR), which is a different peptide and may not retain the inhibitory effect observed previously [82].

Some previous studies have found that TAT-tagged proteins can remain trapped in endosomes. For example, studies of TAT-tagged green fluorescent protein (GFP) indicated inefficient endosomal escape at a concentration of 10 µM [83], yet we operated at a concentration 10 to 20-folder lower with TAT-tagged AKT1 (Figure S5). The same study [83] found that fusion of a haemagglutinin (HA) tag produced a TAT-HA-GFP that was well-distributed in the cytosol according to confocal imaging. According to fluorescence microscopy and confocal imaging experiments, our TAT-AKT1 variants showed similar cytosolic distribution to the TAT-HA-GFP previously reported [83] without the need of an additional HA tag. Thus, the nature of the protein cargo is a critical factor to achieve cytosolic delivery of TAT-tagged proteins [84]. It is also well-established that in the absence of inhibitors of endosomal release, TAT-tagged proteins are released from endosomes and delivered to the cytosol and nucleus [85].

To our knowledge, ours is the first report of fusing the TAT peptide to a full-length and active human kinase and successful demonstration of delivering a recombinant human kinase to human cells. Here, we generated novel TAT-tagged AKT1 and pAKT1 proteins using an approach that we previously developed to generate differentially active variants of full-length human AKT1 from *E. coli* [33]. The Thr308 phosphorylation is achieved by co-expression of AKT1 with the natural upstream Thr308 kinase PDK1 in *E. coli*. While we [19,32,34] and others [61] have also used this approach to produce a truncated AKT1 lacking the N-terminal PH domain, here and previously [33] we found this same approach works well to generate full-length AKT1 containing stoichiometric phosphorylation at Thr308. In our previous studies [19,31,32,33,34], we combined this approach with genetic code expansion to incorporate pSer473 in response to a UAG stop codon at the second regulatory site in AKT1, and thus produced AKT1 with phosphorylation at either or both regulatory sites. We also showed that pThr308 is necessary and sufficient to stimulate maximal AKT1 signaling in mammalian cells [33], and we employed a large scale chemoproteomic approach to determine that pSer473 functions to tune the substrate selectivity of AKT1 [19,32]. In future and on-going studies, beyond the scope of the current work, we are generating TAT-tagged version of AKT1 with phosphorylation at only Ser473 or at both activating sites.

## 5. Conclusions

We demonstrated a novel approach using a TAT fusion peptide to deliver both inactive and activated versions of the human kinase AKT1 to human cells. In our future work with this approach, we will be able to deliver different phospho-AKT1 variants to mammalian cells and study their potential to activate AKT1 signaling and to tune AKT1 substrate selectivity in cells. We anticipate that our approach will serve as a blueprint to deliver other isoforms and phospho-forms of AKT to human cells as well as a wide variety of other human kinases to provide novel methods to study the human kinome in the homologous context of human cells.

## Data Availability

All data are available in the Figures and Supplementary Material.

## Supplementary Materials

The following supporting information can be downloaded at: https://www.mdpi.com/article/10.3390/cells11233834/s1, S.1 Supplementary Figures: Figure S1. Purification of AKT1 and TAT-AKT1 variants; Figure S2. Immunoblotting of purified AKT1 and TAT-AKT1 variants and mass spectrometry of TAT-pAKT1^T308^; Figure S3. Autoradiography images of enzymatic activity assays of AKT1 and TAT-AKT1 variants; Figure S4. AKT1 and pAKT1^T308^ lacking the TAT-tag were not delivered to the interior of HEK 293T cells; Figure S5. Confocal images showing localization of TAT-AKT1 and TAT-pAKT1^T308^ in HEK 293T cells; Figure S6. TAT-AKT1 delivery is not toxic to HEK 293T cells. S.2 TAT-tagged DNA and protein sequences for AKT1 constructs; S.3 Supplementary Methods.
