# Supplementary material for: Delivery of Active AKT1 to Human Cells
Source: Cells. 2022 Nov 29;11(23):3834. doi: 10.3390/cells11233834 (PMC9738475; doi:10.3390/cells11233834)
Supplement: Supplementary file 1 [file cells-11-03834-s001.zip › cells-2009952-supplementary.pdf]

Supplementary Material for:

Delivery of active AKT1 to human cells

Tarana Siddika[1], Nileeka Balasuriya[1], Mallory I. Frederick[1], Peter Rozik[1], Ilka U. Heinemann[1,*], & Patrick O'Donoghue[1,2,*]

[1]Departments of Biochemistry and [2]Chemistry, The University of Western Ontario, London, Ontario, Canada, N6A 5C1

*Correspondence: ilka.heinemann@uwo.ca; patrick.odonoghue@uwo.ca

S.1 Supplementary Figures

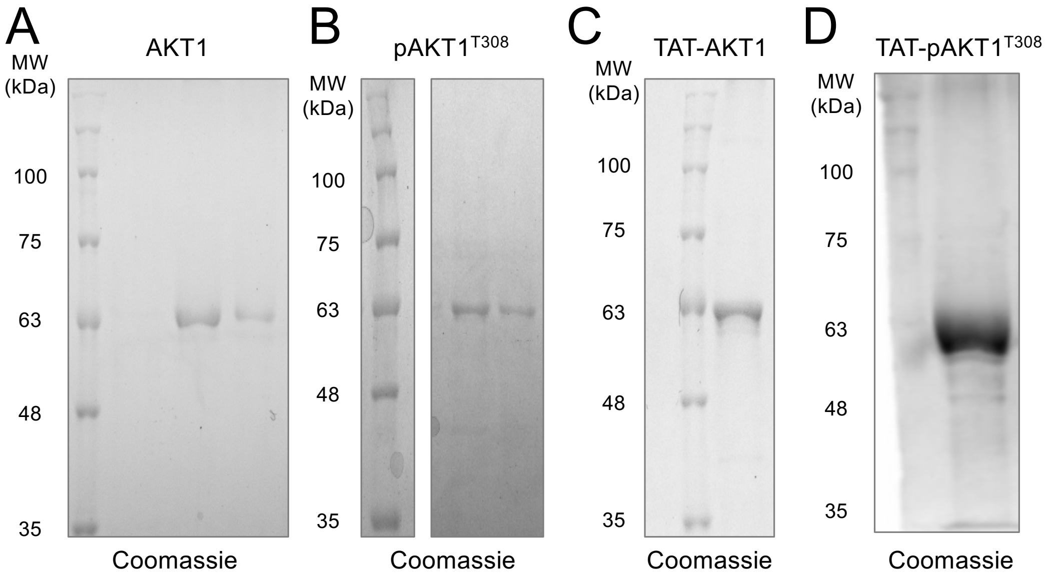

Figure S1. Purification of AKT1 and TAT-AKT1 variants. AKT1 variants (A) AKT1, (B) pAKT1^{T308}, (C) TAT-AKT1, and (D) TAT-pAKT1^{T308} were purified (see Methods), separated by SDS-PAGE, and visualized by Coomassie blue staining.

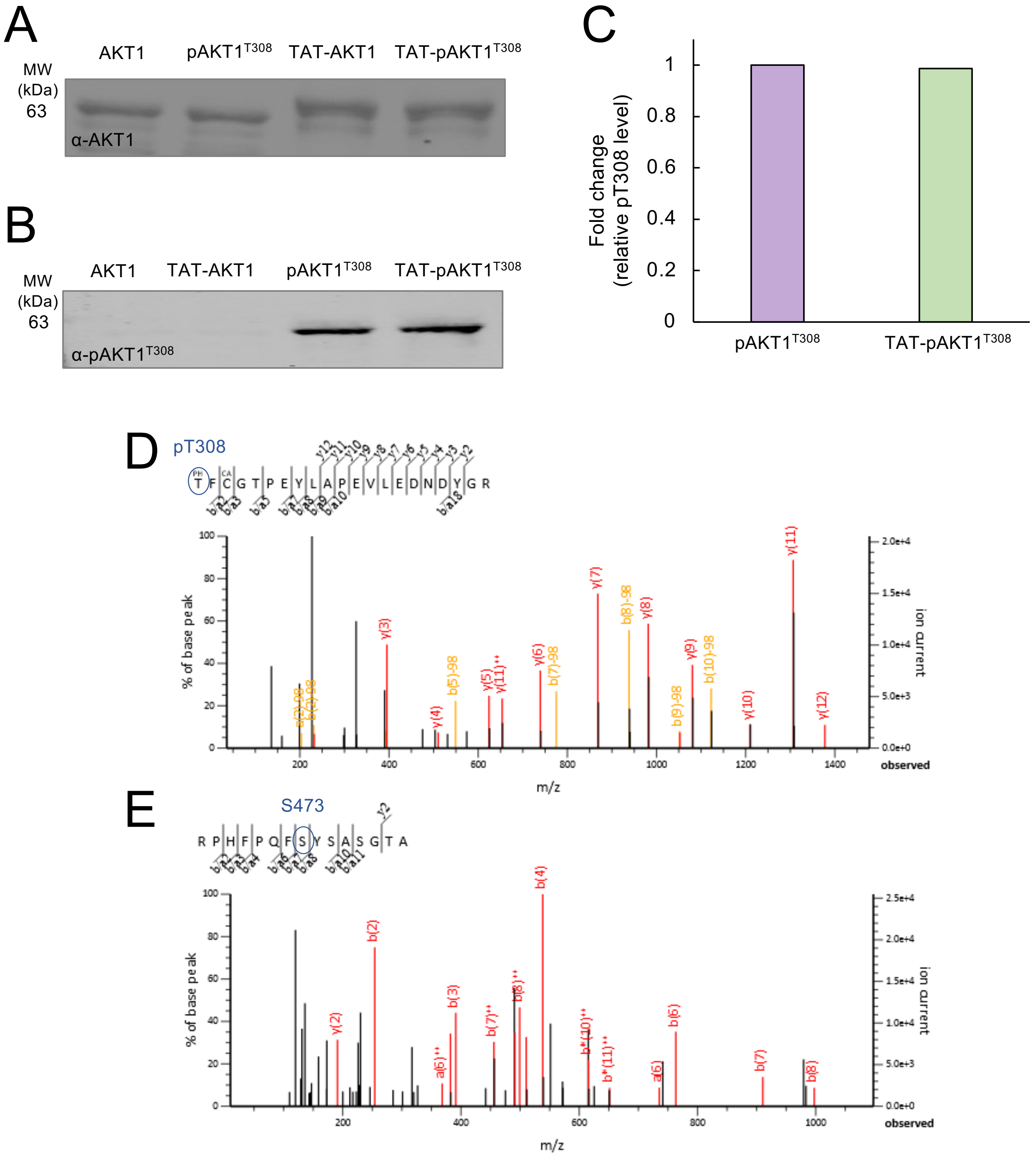

Figure S2. Immunoblotting of purified AKT1 and TAT-AKT1 variants and mass spectrometry of TAT-pAKT1^{T308}. (A) Purified AKT1, pAKT1^{T308}, TAT-AKT1, and TAT-pAKT1^{T308} were separated by SDS-page and immunoblotted with AKT1 specific antibodies. In an independent western blot (B) purified AKT1, TAT-AKT1, pAKT1^{T308}, and TAT-pAKT1^{T308} were visualized with a pAKT^{T308} specific antibodies, demonstrating Thr308 phosphorylation of pAKT1^{T308} and TAT-pAKT1^{T308}, but not TAT-AKT1 or AKT1. (C) Quantification of the blots shows an equivalent level of phosphorylation in pAKT1^{T308} and TAT-pAKT1^{T308}. LC-MS/MS of the purified TAT-pAKT1^{T308} confirmed identification of peptides including (D) pThr308 and (E) Ser473 that demonstrates phosphorylation at Thr308 with no evidence of phosphorylation at Ser473.

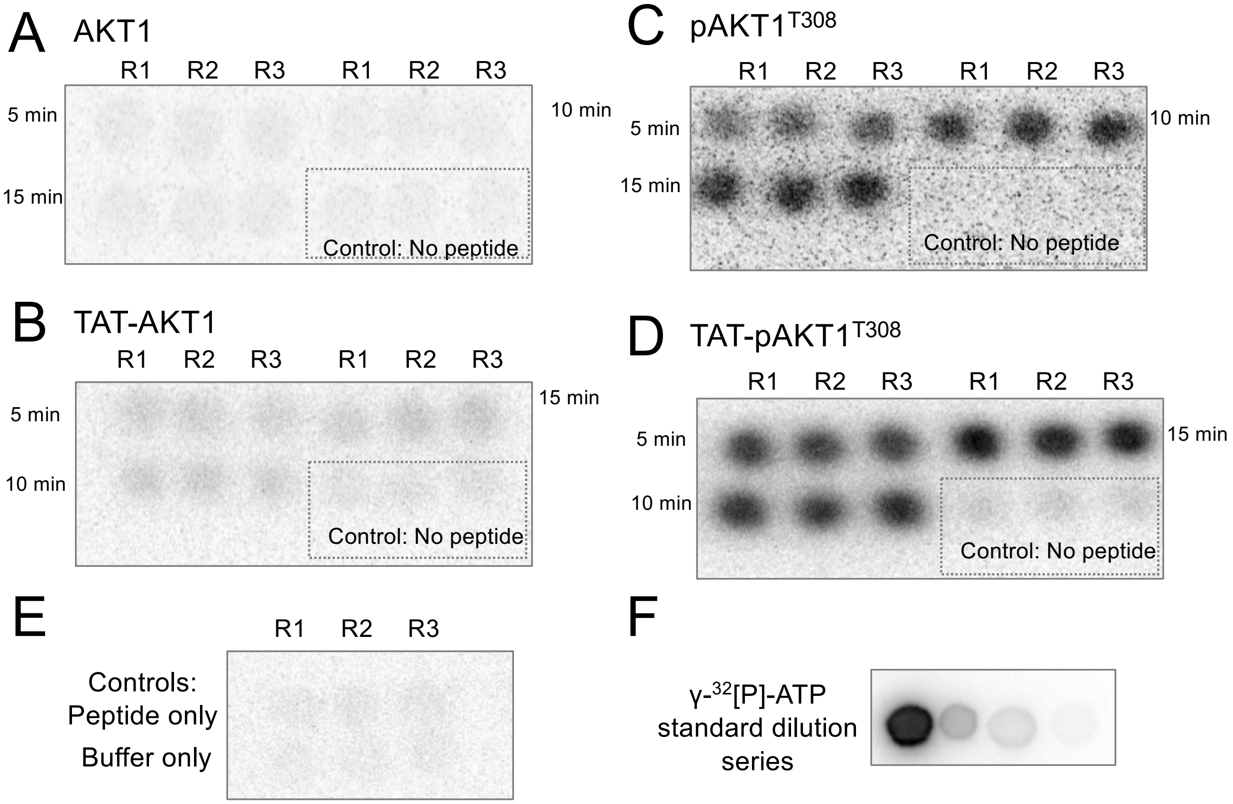

Figure S3. Autoradiography images of enzymatic activity assays of AKT1 and TAT-AKT1 variants. AKT1 variants were incubated with a GSK-3β substrate peptide and [γ-^{32}P]-ATP and spotted on filter paper. Unreacted [γ-^{32}P]-ATP was washed away and reaction products were visualized by phosphorimaging. Images of three independent enzyme reactions of (A) AKT1, (B) TAT-AKT1, (C) pAKT1^{T308}, (D) TAT-pAKT1^{T308}, and (E) no enzyme controls of peptide only (above) and buffer only (below). (F) The amount of phosphorylated peptide produced quantified using a [γ-^{32}P]-ATP dilution series.

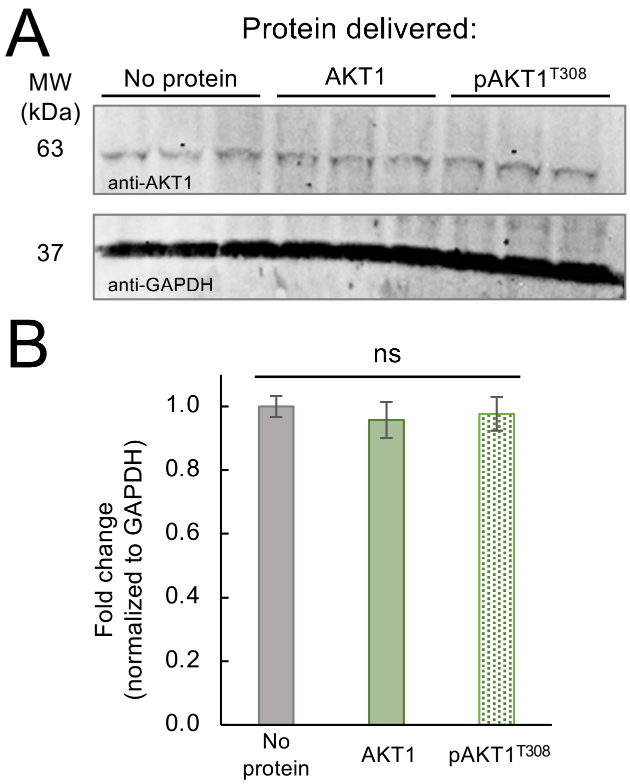

Figure S4. AKT1 and pAKT1^{T308} lacking the TAT-tag were not delivered to the interior of HEK 293T cells. HEK 293T cells were incubated with no protein, purified AKT1, or pAKT1^{T308} for 1 hour in three biological replicates. (A) Western blots and (B) quantification of cells extracts separated by SDS-PAGE and probed with AKT1 and GAPDH specific antibodies. Endogenous AKT1 was detected by immunoblotting and no increase in the AKT1 signal was observed after incubation with AKT1 variants lacking the TAT-tag. P-values were calculated by one-way ANOVA and are indicated (ns – not significant).

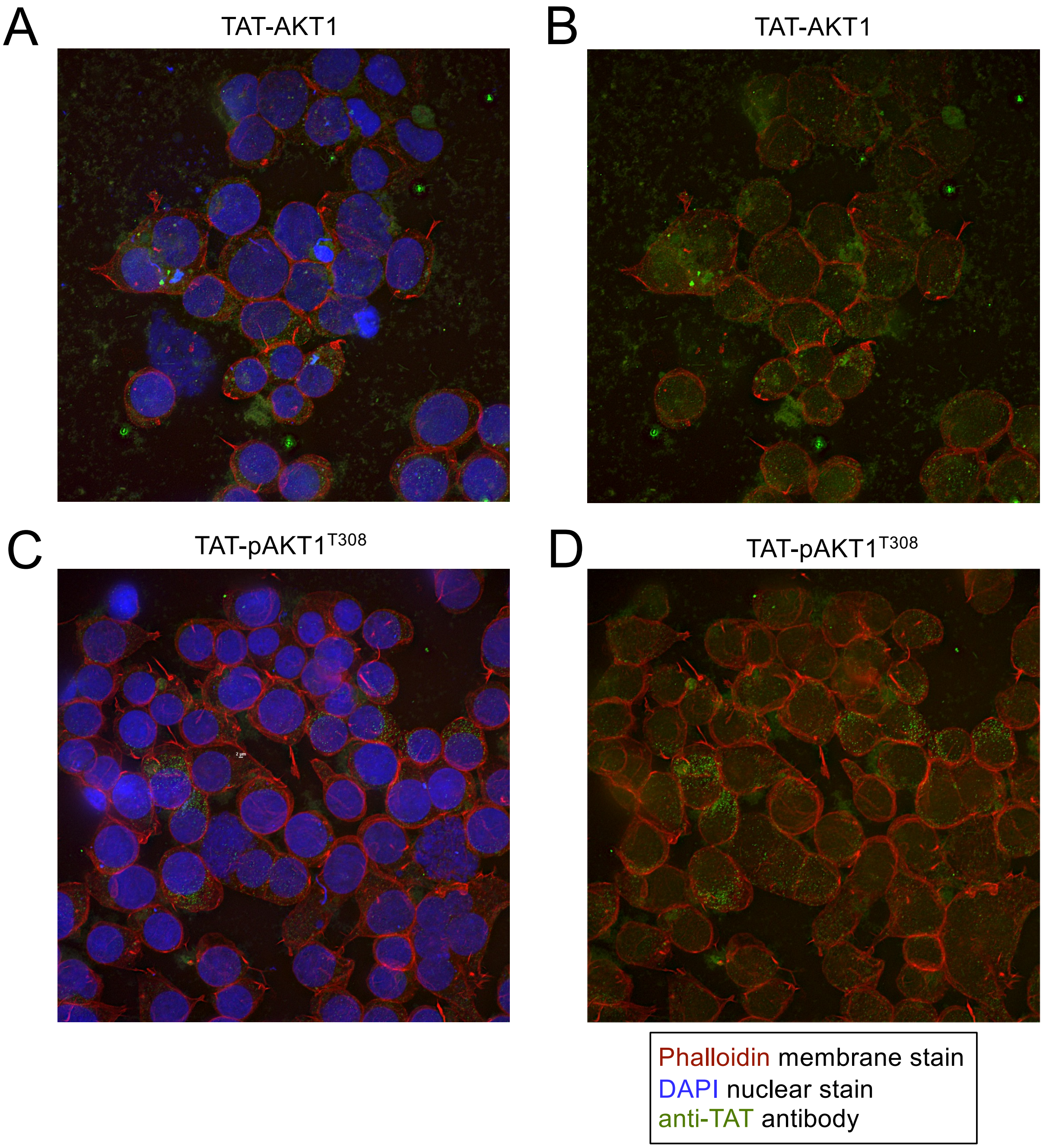

Figure S5. Confocal images showing localization of TAT-AKT1 and TAT-pAKT1^{T308} in HEK 293T cells. HEK 293T cells were incubated with (A,B) TAT-AKT1 or (C,D) TAT-pAKT1^{T308}. To detect TAT-tagged AKT1 variants delivered to the cells, confocal microscopy and immunofluorescence was used with an HIV-TAT-antibody and AlexaFluor568-linked secondary antibody (green). The cell nuclei were stained with DAPI (blue), and cell membranes were stained with AlexaFluor647-linked phalloidin (red). Confocal images show (A, C) overlay of anti-TAT, DAPI, and phalloidin, and (B, D) overlay of anti-TAT and phalloidin. The images show that both TAT-AKT1 and TAT-pAKT1^{T308} are well-distributed inside of the cells.

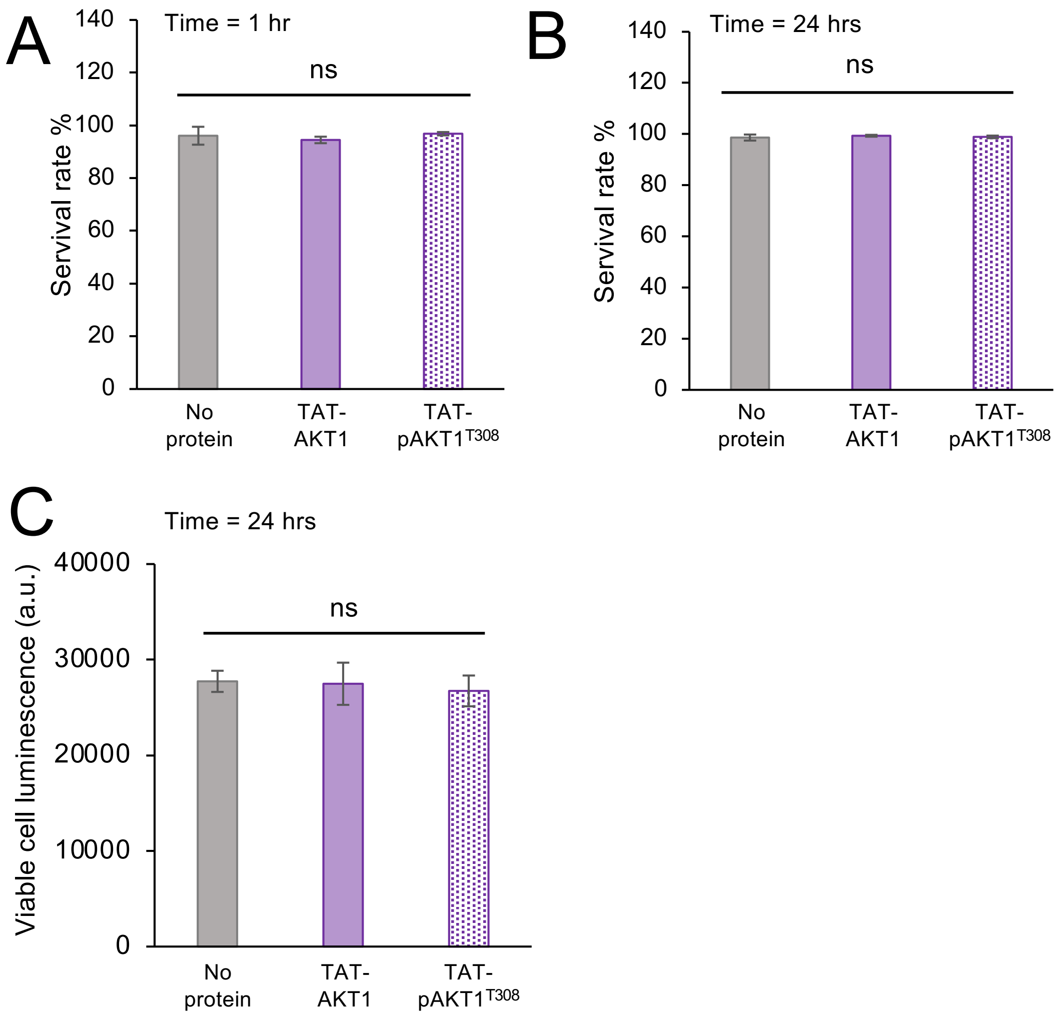

Figure S6. TAT-AKT1 delivery is not toxic to HEK 293T cells. Cell viability and survival were measured in two different assays: (A, B) trypan blue assay and (C) CytoTox-Glo assay. Survival rates were obtained by staining dead cells after incubating HEK 293T cells with no protein, TAT-AKT1, or TAT-pAKT1^{T308}. After (A) 1 hour or (B) 24 hours of incubation and subsequent staining with trypan blue for dead cells, cells were imaged, and the relative number of dead cells quantified. No significant difference in cell survival was observed. (C) After 24 hours of incubation without protein or with TAT-AKT1 or TAT-pAKT1^{T308}, CytoTox-Glo was added to the cells and luminescence from dead-cell protease activity was recorded using a Synergy H1 Plate reader. P-values were calculated by one-way ANOVA and are indicated (ns – not significant).

S.2 TAT-tagged DNA and protein sequences for AKT1 constructs

His$_6$-TAT-AKT1 DNA construct (codon optimized for *E. coli)*

CCATGGATGCGGGGTTCTCATCATCATCATCATCATGGTATGGCTAGCATGACTGGTGGACAGCAAATGGGTCGGGA
TCTGTACGACGATGACGATAAGGATCGATGGGGTTCTAAGCTTGGCTACGGCCGCAAGAAACGCCGCCAGCGCCGCC
GCGGTGGATCCACCATGTCTGACGTTGCTATCGTTAAAGAAGGTTGGCTGCACAAACGTGGTGAATACATCAAAACC
TGGCGTCCGCGTTACTTCCTGCTGAAAAACGACGGTACCTTCATCGGTTACAAAGAACGTCCGCAGGACGTTGACCA
GCGTGAAGCTCCGCTGAACAACTTCTCTGTTGCTCAGTGCCAGCTGATGAAAACCGAACGTCCGCGTCCGAACACCT
TCATCATCCGTTGCCTGCAGTGGACCACCGTTATCGAACGTACCTTCCACGTTGAAACCCCGGAAGAACGTGAAGAA
TGGACCACCGCTATCCAGACCGTTGCTGACGGTCTGAAAAAACAGGAAGAAGAAGAAATGGACTTCCGTTCTGGTTC
TCCGTCTGACAACTCTGGTGCTGAAGAAATGGAAGTTTCTCTGGCTAAACCGAAACACCGTGTTACCATGAACGAAT
TTGAATACCTGAAACTGCTGGGTAAAGGTACCTTCGGTAAAGTTATCCTGGTTAAAGAAAAAGCTACCGGTCGTTAC
TACGCTATGAAAATCCTGAAAAAAGAAGTTATCGTTGCTAAAGACGAAGTTGCTCACACCCTGACCGAAAACCGTGT
TCTGCAGAACTCTCGTCACCCGTTCCTGACCGCTCTGAAATACTCTTTCCAGACCCACGACCGTCTGTGCTTCGTTA
TGGAATACGCTAACGGTGGTGAACTGTTCTTCCACCTGTCTCGTGAACGTGTTTTCTCTGAAGACCGTGCTCGTTTC
TACGGTGCTGAAATCGTTTCTGCTCTGGACTACCTGCACTCTGAAAAAAACGTTGTTTACCGTGACCTGAAACTGGA
AAACCTGATGCTGGACAAAGACGGTCACATCAAAATCACCGACTTCGGTCTGTGCAAAGAAGGTATCAAAGACGGTG
CTACCATGAAAACCTTCTGCGGTACCCCGGAATACCTGGCTCCGGAAGTTCTGGAAGACAACGACTACGGTCGTGCT
GTTGACTGGTGGGGTCTGGGTGTTGTTATGTACGAAATGATGTGCGGTCGTCTGCCGTTCTACAACCAGGACCACGA
AAAACTGTTCGAACTGATCCTGATGGAAGAAATCCGTTTCCCGCGTACCCTGGGTCCGGAAGCTAAATCTCTGCTGT
CTGGTCTGCTGAAAAAAGACCCGAAACAGCGTCTGGGTGGTGGTTCTGAAGACGCTAAAGAAATCATGCAGCACCGT
TTCTTCGCTGGTATCGTTTGGCAGCACGTTTACGAAAAAAAACTGTCTCCGCCGTTCAAACCGCAGGTTACCTCTGA
AACCGACACCCGTTACTTCGACGAAGAATTTACCGCTCAGATGATCACCATCACCCCGCCGGACCAGGACGACTCTA
TGGAATGCGTTGACTCTGAACGTCGTCCGCACTTCCCGCAGTTCTCTTACTCTGCTTCTGGTACCGCTTAAGCGGCC
GC

CCATGG – *Nco*I
CGGCCGC – *Not*I
GGATCC – *Bam*HI
His6 tag
TAT DNA sequence
AKT1 sequence, codon optimized (purple lettering)

His$_6$-TAT-AKT1 protein sequence

MRGSHHHHHHGMASMTGGQQMGRDLYDDDDKDRWGSKLGYGRKKRRQRRRGGSTMSDVAIVKEGWLHKRGEYIKTWR
PRYFLLKNDGTFIGYKERPQDVDQREAPLNNFSVAQCQLMKTERPRPNTFIIRCLQWTTVIERTFHVETPEEREEWT
TAIQTVADGLKKQEEEEMDFRSGSPSDNSGAEEMEVSLAKPKHRVTMNEFEYLKLLGKGTFGKVILVKEKATGRYYA
MKILKKEVIVAKDEVAHTLTENRVLQNSRHPFLTALKYSFQTHDRLCFVMEYANGGELFFHLSRERVFSEDRARFYG
AEIVSALDYLHSEKNVVYRDLKLENLMLDKDGHIKITDFGLCKEGIKDGATMKTFCGTPEYLAPEVLEDNDYGRAVD
WWGLGVVMYEMMCGRLPFYNQDHEKLFELILMEEIRFPRTLGPEAKSLLSGLLKKDPKQRLGGGSEDAKEIMQHRFF
AGIVWQHVYEKKLSPPFKPQVTSETDTRYFDEEFTAQMITITPPDQDDSMECVDSERRPHFPQFSYSASGTA

HHHHHH – His6 tag
YGRKKRRQRRR – TAT peptide sequence
AKT1 sequence (purple lettering)

His$_6$-TAT-mCherry-AKT1 DNA constructs (codon optimized for *E. coli)*

CCATGGATGCGGGGTTCTCATCATCATCATCATCATGGTATGGCTAGCATGACTGGTGGACAGCAAATGGGTCGGGA
TCTGTACGACGATGACGATAAGGATCGATGGGGTTCTAAGCTTGGCTACGGCCGCAAGAAACGCCGCCAGCGCCGCC
GCGGTGGATCCACTATGGTGTCGAAGGGCGAGGAGGACAACATGGCCATCATCAAGGAGTTCATGCGCTTCAAGGTG
CACATGGAGGGCTCCGTGAACGGCCACGAGTTCGAGATCGAGGGCGAGGGCGAGGGCCGCCCCTACGAGGGCACCCA
GACCGCCAAGCTGAAGGTGACCAAGGGTGGCCCCCTGCCCTTCGCCTGGGACATCCTGTCCCCTCAGTTCATGTACG
GCTCCAAGGCCTACGTGAAGCACCCCGCCGACATCCCCGACTACTTGAAGCTGTCCTTCCCCGAGGGCTTCAAGTGG
GAGCGCGTGATGAACTTCGAGGACGGCGGCGTGGTGACCGTGACCCAGGACTCCTCCCTGCAGGACGGCGAGTTCAT
CTACAAGGTGAAGCTGCGCGGCACCAACTTCCCCTCCGACGGCCCCGTAATGCAGAAGAAGACTATGGGTTGGGAGG
CCTCCTCCGAGCGGATGTACCCCGAGGACGGCGCCCTGAAGGGCGAGATCAAGCAGAGGCTGAAGCTGAAGGACGGC
GGCCACTACGACGCTGAGGTCAAGACCACCTACAAGGCCAAGAAGCCCGTGCAGCTGCCCGGCGCCTACAACGTCAA
CATCAAGTTGGACATCACCTCCCACAACGAGGACTACACCATCGTGGAACAGTACGAACGCGCCGAGGGCCGCCACT
CCACCGGCGGCATGGACGAGCTGTATAAGGGCACTGAACTGGGGAATTCCTCTGACGTTGCTATCGTTAAAGAAGGT
TGGCTGCACAAACGTGGTGAATACATCAAAACCTGGCGTCCGCGTTACTTCCTGCTGAAAAACGACGGTACCTTCAT
CGGTTACAAAGAACGTCCGCAGGACGTTGACCAGCGTGAAGCTCCGCTGAACAACTTCTCTGTTGCTCAGTGCCAGC
TGATGAAAACCGAACGTCCGCGTCCGAACACCTTCATCATCCGTTGCCTGCAGTGGACCACCGTTATCGAACGTACC
TTCCACGTTGAAACCCCGGAAGAACGTGAAGAATGGACCACCGCTATCCAGACCGTTGCTGACGGTCTGAAAAAACA
GGAAGAAGAAGAAATGGACTTCCGTTCTGGTTCTCCGTCTGACAACTCTGGTGCTGAAGAAATGGAAGTTTCTCTGG
CTAAACCGAAACACCGTGTTACCATGAACGAATTTGAATACCTGAAACTGCTGGGTAAAGGTACCTTCGGTAAAGTT
ATCCTGGTTAAAGAAAAAGCTACCGGTCGTTACTACGCTATGAAAATCCTGAAAAAAGAAGTTATCGTTGCTAAAGA
CGAAGTTGCTCACACCCTGACCGAAAACCGTGTTCTGCAGAACTCTCGTCACCCGTTCCTGACCGCTCTGAAATACT
CTTTCCAGACCCACGACCGTCTGTGCTTCGTTATGGAATACGCTAACGGTGGTGAACTGTTCTTCCACCTGTCTCGT
GAACGTGTTTTCTCTGAAGACCGTGCTCGTTTCTACGGTGCTGAAATCGTTTCTGCTCTGGACTACCTGCACTCTGA
AAAAAACGTTGTTTACCGTGACCTGAAACTGGAAAACCTGATGCTGGACAAAGACGGTCACATCAAAATCACCGACT
TCGGTCTGTGCAAAGAAGGTATCAAAGACGGTGCTACCATGAAAACCTTCTGCGGTACCCCGGAATACCTGGCTCCG
GAAGTTCTGGAAGACAACGACTACGGTCGTGCTGTTGACTGGTGGGGTCTGGGTGTTGTTATGTACGAAATGATGTG
CGGTCGTCTGCCGTTCTACAACCAGGACCACGAAAAACTGTTCGAACTGATCCTGATGGAAGAAATCCGTTTCCCGC
GTACCCTGGGTCCGGAAGCTAAATCTCTGCTGTCTGGTCTGCTGAAAAAAGACCCGAAACAGCGTCTGGGTGGTGGT
TCTGAAGACGCTAAAGAAATCATGCAGCACCGTTTCTTCGCTGGTATCGTTTGGCAGCACGTTTACGAAAAAAAACT
GTCTCCGCCGTTCAAACCGCAGGTTACCTCTGAAACCGACACCCGTTACTTCGACGAAGAATTTACCGCTCAGATGA
TCACCATCACCCCGCCGGACCAGGACGACTCTATGGAATGCGTTGACTCTGAACGTCGTCCGCACTTCCCGCAGTTC
TCTTACTCTGCTTCTGGTACCGCTTAAGCGGCCGC

CCATGG – *Nco*I
CGGCCGC – *Not*I
GGATCC – *Bam*HI
GAATTC – *Eco*RI
His6 tag
TAT DNA sequence
mCherry sequence (red lettering)
Linker sequence (grey lettering)
AKT1 sequence, codon optimized (purple lettering)

His_6-TAT-mCherry-AKT1 protein sequence

MRGSHHHHHHGMASMTGGQQMGRDLYDDDDKDRWGSKLGYGRKKRRQRRRGGSTMVSKGEEDNMAIIKEFMRFKVHM
EGSVNGHEFEIEGEGEGRPYEGTQTAKLKVTKGGPLPFAWDILSPQFMYGSKAYVKHPADIPDYLKLSFPEGFKWER
VMNFEDGGVVTVTQDSSLQDGEFIYKVKLRGTNFPSDGPVMQKKTMGWEASSERMYPEDGALKGEIKQRLKLKDGGH
YDAEVKTTYKAKKPVQLPGAYNVNIKLDITSHNEDYTIVEQYERAEGRHSTGGMDELYKGTELGNSSDVAIVKEGWL
HKRGEYIKTWRPRYFLLKNDGTFIGYKERPQDVDQREAPLNNFSVAQCQLMKTERPRPNTFIIRCLQWTTVIERTFH
VETPEEREEWTTAIQTVADGLKKQEEEEMDFRSGSPSDNSGAEEMEVSLAKPKHRVTMNEFEYLKLLGKGTFGKVIL
VKEKATGRYYAMKILKKEVIVAKDEVAHTLTENRVLQNSRHPFLTALKYSFQTHDRLCFVMEYANGGELFFHLSRER
VFSEDRARFYGAEIVSALDYLHSEKNVVYRDLKLENLMLDKDGHIKITDFGLCKEGIKDGATMKTFCGTPEYLAPEV
LEDNDYGRAVDWWGLGVVMYEMMCGRLPFYNQDHEKLFELILMEEIRFPRTLGPEAKSLLSGLLKKDPKQRLGGGSE
DAKEIMQHRFFAGIVWQHVYEKKLSPPFKPQVTSETDTRYFDEEFTAQMITITPPDQDDSMECVDSERRPHFPQFSY
SASGTA

HHHHHH – His6 tag
YGRKKRRQRRR – TAT peptide sequence
mCherry sequence (red lettering)
Linker sequence (grey lettering)
AKT1 sequence (purple lettering)

S.3 Supplementary Methods

TAT-AKT localization, confocal imaging, and immunofluorescence. HEK 293T cells were cultured in T25 flask containing DMEM (Cellgro, ThermoFisher Scientific, Ottawa, ON) supplemented with 10% fetal bovine serum and 1% penicillin/streptomycin at 37 °C with the supply of 5% CO_2. Poly-D-lysine coated cover slips were prepared for cell attachment. In the fume hood, a monolayer of clean coverslips was submerged in 4 M HCl at 60 °C for 3 hours and the amount of HCl added was sufficient to submerge the coverslips. After 3 hours, the coverslips were washed several times with sterile and double distilled H_2O (ddH_2O). The coverslips were placed into 70% ethanol for storage. Prior to use, each coverslip was cleaned with Kimwipes to remove contaminants and dried in the Biological Safety Cabinet (BSC) for 30 minutes. Then each coverslip was placed in a 6-well dish and exposed to ultra-violet light for 1 hr. To coat the coverslips, poly-D-lysine was diluted in sterile ddH_2O to 50 μg/mL and added to a volume of 0.5 mL in each well and incubated for 2 hours at room temperature in a BSC. The excess poly-D-lysine was removed, and coverslips were dried in a BSC for 2 hours.

To attach the cells to the coated coverslip, 50,000 cell/mL were plated in each well containing DMEM media with 10% fetal bovine serum and 1% penicillin/streptomycin. Cells were kept at 37 °C with the supply of 5% CO_2. After 2 days, 0.5 μM of TAT-AKT1 or TAT-pAKT1^{T308} protein were added and incubated for 24 hours. The cover slips were then washed 2 × 5 minutes with phosphate buffered solution (Corning cellgro, #21-040-CM). To fix the cells, 1 mL of 4% (v/v) paraformaldehyde diluted in phosphate buffered solution was added to each well and incubated for 30 minutes at room temperature. The coverslips were then rinsed 3 × 5 minutes with phosphate buffered saline (PBS). To permeabilize the cell membrane, 1 mL of 0.2% (v/v) Tween20 in PBS was added to each well and incubated for 10 minutes and rinsed with PBS 2 × 5 minutes. A volume of 50 μL of Background Sniper blocking reagent (Biocare Medical, Pacheco, CA, USA, #BS966L) was added to each coverslip and incubated for 7 minutes at room temperature.

For immunofluorescence, the primary anti-HIV1 TAT antibody [N3] (Abcam, #ab63957) was diluted (1:500) into Dako Ab Diluent (Agilent Technologies, Santa Clara, CA, USA, #S0809) and 50 μL was applied to each coverslip and incubated overnight at 4 °C. The coverslips were washed with PBS 3 × 5 minutes. The

secondary antibody Goat anti-Mouse IgG (H+L) Highly Cross-Adsorbed Secondary Antibody, AlexaFluor568 (568 nm excitation) (ThermoFisher Scientific, #A11031) was diluted in Dako Ab Diluent and 50 μL was applied to each coverslip and incubated for 30 minutes at room temperature. The coverslips were rinsed 2 × 5 minutes with PBS. The cell membrane stain, AlexaFluor647-Phalloidin (647 nm excitation) (ThermoFisher Scientific, #A22287), was diluted (1:250) in PBS, 50 μL was added to each coverslip, and incubated for 15 minutes at room temperature. The coverslips were rinsed 2 × 5 minutes with PBS. The 4′,6-diamidino-2-phenylindole (DAPI) (ThermoFisher Scientific, #D1306) nuclear counterstain was diluted (1:300) in PBS and 50 μL was added to each coverslip and incubated for 2 minutes at room temperature. The coverslips were washed 2 × 5 minutes with PBS. A drop of anti-faded mountant (Prolong gold) was added to each slide and each coverslip was placed upside down on the mountant. The coverslips were then stored in the dark at room temperature for 24 hours and excess mountant was wiped from the edges of the coverslips, which were then sealed with nail polish and stored at 4 °C for further analysis. Images of cells from each coverslip was taken with a Nikon Inverted T21E Deconvolution microscope (Nikon Metrology, Inc, Brighton, MI, USA). Images were captured with a Nikon 60X objective (Plan Apochomat lambda oil, 1.4 numerical aperture/0.13 mm working distance, DIC). Fluorescence images were acquired with the indicated filter configurations to detect AlexaFlour-647 phalloidin (excitation 635 ± 22 nm, emission 680 ± 42 nm), the anti-TAT secondary antibody conjugated to AlexaFluor-568 (excitation 555 ± 28 nm, emission 595 ± 31 nm), and DAPI (excitation 375 ± 30 nm, emission 432 ± 36 nm).
